# Altered transcription factor targeting is associated with differential peripheral blood mononuclear cell proportions in sarcoidosis

**DOI:** 10.3389/fimmu.2022.848759

**Published:** 2022-10-13

**Authors:** Christian Ascoli, Cody A. Schott, Yue Huang, Benjamin A. Turturice, Wangfei Wang, Naomi Ecanow, Nadera J. Sweiss, David L. Perkins, Patricia W. Finn

**Affiliations:** ^1^ Division of Pulmonary, Critical Care, Sleep, and Allergy, Department of Medicine, University of Illinois at Chicago, Chicago, IL, United States; ^2^ University of Illinois at Chicago College of Medicine, Chicago, IL, United States; ^3^ Department of Bioengineering, University of Illinois at Chicago College of Engineering and Medicine, Chicago, IL, United States; ^4^ Division of Rheumatology, Department of Medicine, University of Illinois at Chicago, Chicago, IL, United States; ^5^ Division of Nephrology, Department of Medicine, University of Illinois at Chicago, Chicago, IL, United States

**Keywords:** sarcoidosis, transcriptomics, immune dysregulation, master regulators, PBMC

## Abstract

**Introduction:**

In sarcoidosis, peripheral lymphopenia and anergy have been associated with increased inflammation and maladaptive immune activity, likely promoting development of chronic and progressive disease. However, the molecular mechanisms that lead to reduced lymphocyte proportions, particularly CD4+ T-cells, have not been fully elucidated. We posit that paradoxical peripheral lymphopenia is characterized by a dysregulated transcriptomic network associated with cell function and fate that results from altered transcription factor targeting activity.

**Methods:**

Messenger RNA-sequencing (mRNA-seq) was performed on peripheral blood mononuclear cells (PBMCs) from ACCESS study subjects with sarcoidosis and matched controls and findings validated on a sarcoidosis case-control cohort and a sarcoidosis case series. Preserved PBMC transcriptomic networks between case-control cohorts were assessed to establish cellular associations with gene modules and define regulatory targeting involved in sarcoidosis immune dysregulation utilizing weighted gene co-expression network analysis and differential transcription factor involvement analysis. Network centrality measures identified master transcriptional regulators of subnetworks related to cell proliferation and death. Predictive models of differential PBMC proportions constructed from ACCESS target gene expression corroborated the relationship between aberrant transcription factor regulatory activity and imputed and clinical PBMC populations in the validation cohorts.

**Results:**

We identified two unique and preserved gene modules significantly associated with sarcoidosis immune dysregulation. Strikingly, increased expression of a monocyte-driven, and not a lymphocyte-driven, gene module related to innate immunity and cell death was the best predictor of peripheral CD4+ T-cell proportions. Within the gene network of this monocyte-driven module, TLE3 and CBX8 were determined to be master regulators of the cell death subnetwork. A core gene signature of differentially over-expressed target genes of TLE3 and CBX8 involved in cellular communication and immune response regulation accurately predicted imputed and clinical monocyte expansion and CD4+ T-cell depletion.

**Conclusions:**

Altered transcriptional regulation associated with aberrant gene expression of a monocyte-driven transcriptional network likely influences lymphocyte function and survival. Although further investigation is warranted, this indicates that crosstalk between hyperactive monocytes and lymphocytes may instigate peripheral lymphopenia and underlie sarcoidosis immune dysregulation and pathogenesis. Future therapies selectively targeting master regulators, or their targets, may mitigate dysregulated immune processes in sarcoidosis and disease progression.

## Introduction

Sarcoidosis is a systemic inflammatory disease characterized by dysregulated immune processes (1). The interactions between lymphocytes, macrophages, and immune mediators, such as tumor necrosis factor alpha (TNF-α) and interferon gamma (IFN-γ), lead to an exaggerated and polarized CD4+ T-helper (Th) 1 and Th17 cell response against an unknown antigenic stimulus at sites of granuloma formation (2). While local CD4+ T-cell accumulation, proliferation, and activation are invariably observed within affected tissues, 10-50% of those with sarcoidosis paradoxically exhibit peripheral lymphopenia coupled with anergy or exhaustion (3–8). When present, lymphopenia specifically related to low CD4+ T-cell counts, has been linked with greater inflammatory activity in sarcoidosis (7).

Historically, lymphopenia has been considered the product of hypersplenism, bone marrow infiltration, or T-cell sequestration (4, 9–11). However, a current paradigm posits that peripheral depletion of lymphocytes results from an imbalance between immunoregulatory and effector T-cell numbers and function in response to prolonged antigenic exposure (6, 8, 12–14). This notion is supported by conventional studies that demonstrate aberrant expression of CD28, CD95 (Fas), CD274 (PD-1), and other molecules linked to programmed cell death and points towards inherent molecular mechanisms contributing to T-cell anergy or exhaustion and development of lymphopenia in sarcoidosis (4, 6–8, 11, 14–17). Accordingly, lymphopenia and loss of effector function is thought to impair immune surveillance and inhibit downregulation of immune reactions thereby eliciting a state of persistent maladaptive inflammatory activity that, in turn, prevents disease resolution and promotes development of chronic disease (4, 7, 13, 14, 18–26). As such, reduced CD4+ T-cell counts, suppressed T-cell function, and decreased lymphocyte gene expression are likely to contribute to disease progression and have been associated with disease severity while reversal of exhaustion has been correlated with disease improvement (1, 4, 8, 16, 27).

Despite these implications, the underpinnings of peripheral lymphopenia in sarcoidosis remain unclear and comprehensive assessment of the genetic repertoire associated with this phenomenon has been limited (25). To date, studies utilizing bulk and single-cell high-throughput transcriptomic methods have prioritized identification of discriminatory gene signatures that support the diagnosis and prognosis of sarcoidosis (1, 25, 28–34). Notwithstanding, a wide variety of differentially expressed genes implicated in significantly enriched biologic processes related to cell proliferation and death with limited overlap among the distinct signatures was discovered (1, 25, 28–34). This evidence suggests that dysregulation of an elaborate gene network involved in multiple biologic processes that impair lymphocyte survival, rather than a single gene or process, likely instigates sarcoidosis-related lymphopenia. In this regard, transcriptomic networks that determine cell function and fate in health and disease primarily depend on the activation or repression of genes through the coordinated regulatory activity of transcription factors (35–38). However, the relevance of transcription factors in gene signatures derived from high-throughput transcriptomic differential expression analyses is often overlooked because of their relatively low and stable abundance as well as the inability of these analyses to determine transcription factor interactions with target genes (39–42).

Based on evidence from our prior studies, we hypothesized that a dysregulated transcriptomic network characterizes circulating immune cells in sarcoidosis and aimed to discern master regulator transcription factors of aberrant gene expression to gain insight into the molecular mechanisms underlying paradoxical peripheral lymphopenia in sarcoidosis (1, 16, 43). Utilizing data acquired from whole blood transcriptome shotgun sequencing of messenger RNA (mRNA-seq) we comprehensively assessed the transcriptomic network of peripheral blood mononuclear cells (PBMCs) in a cohort of subjects with sarcoidosis and their matched controls and validated our findings across high-throughput transcriptomic platforms on an independent case-control cohort from the University California at San Francisco (UCSF) and an independent case-series from the University of Illinois at Chicago Bernie Mac Sarcoidosis Translational Advanced Research (UIC STAR) Center. Focusing on biologic processes associated with cell proliferation and death, we implemented a combinatorial systems biology approach that integrated weighted gene co-expression network analysis, differential transcription factor involvement analysis, and cellular deconvolution to define regulatory targeting corresponding to differential expression patterns associated with lymphopenia in sarcoidosis. The transcription factors TLE3 and CBX8 were determined to be master regulators of a monocyte-driven “cell-death” subnetwork and their differentially expressed targets accurately predicted imputed CD14+ monocyte expansion and CD4+ T-cell depletion in the UCSF cohort. Additionally, upon determination of congruency between imputed and clinical cell proportions, the classification performance of TLE3 and CBX8 gene targets was further validated and determined accurately predict monocyte and CD4+ T-cell proportions in the UIC STAR cohort. Altogether, our findings indicate that transcriptomic changes detrimental to lymphocyte function and survival resulting from altered regulation of the monocyte-driven module may instigate peripheral lymphopenia in sarcoidosis through mechanisms involved in regulation of cell-cell communication and immune response activation. Our study adds an additional facet to the understanding of sarcoidosis; crosstalk between hyperactive monocytes and lymphocytes may be driving immune dysregulation and pathogenesis.

## Methods

### Study design and population

Study approval was obtained through the UIC institutional review board (Protocol #s: 2019-0452 and 2016-0063). Briefly, upon messenger RNA extraction from peripheral blood mononuclear cells (PBMCs), gene expression data obtained from whole transcriptome shotgun sequencing (mRNA-seq) was investigated in treatment naïve Caucasian subjects newly diagnosed with sarcoidosis (n=14) and matched controls (n=14). All 28 subjects were enrolled through the RNA core laboratory in “A Case Controlled Etiologic Study of Sarcoidosis” (ACCESS Study, ClinicalTrials.gov Identifier: NCT00005276) as previously described and utilized as a reference cohort in this study given that the gene expression profiles from cases were deemed to represent baseline immune dysregulation in sarcoidosis (1, 44). Inclusion of sarcoidosis cases for the ACCESS study required tissue confirmation of non-caseating granulomas within six months of enrollment and exclusion of other possible causes of granulomatous inflammation. ACCESS study controls were selected by random digit dialing and matched to cases by gender, age, race, and geographic region and excluded if there was a prior history of granulomatous disease or reported use of anti-tuberculosis therapy. Individual subject clinical characteristics were defined and reported per ACCESS study protocols (**Supplemental Table S1A**) (44). To substantiate gene signatures identified from computational analyses implemented on the ACCESS cohort, validation was performed on gene expression data derived from the “UCSF Sarcoidosis and Hypersensitivity Pneumonitis Cohort” (Gene Expression Omnibus [GEO] series record GSE19314, **Supplemental Table S1B**) and the University of Illinois at Chicago Bernie Mac Sarcoidosis Translational Advanced Research (UIC STAR) Center (**Supplemental Table S1C**) (29, 34, 45). The UCSF cohort was selected after performing a systematic search on GEO for publicly available PBMC gene expression data from predominantly Caucasian sarcoidosis case-control cohorts to statistically validate our results on a genotypically similar population. The UIC STAR cohort was selected to further assess the validity of our findings on a genotypically heterogeneous population and to assess the validity of *in-silico* cellular deconvolution. Further details pertaining to study cohorts, mRNA-seq library preparation and annotation for the ACCESS cohort, processing of raw microarray expression data for the UCSF cohort, and mRNA-seq library preparation and annotation for the UIC STAR cohort are described at length in the data supplement. All raw sequencing data for the ACCESS cohort has been deposited to GEO, series record GSE155644. **Supplemental Figure S1** depicts a summary of the workflow of methods used in this study.

### Construction of gene co-expression modules and determination of module biologic function

A signed gene co-expression network for the ACCESS cohort was constructed utilizing weighted gene co-expression network analysis (*WGCNA*) to identify gene modules or clusters and their relationship with sarcoidosis and other clinical features (46, 47). Given differences in RNA sequencing platforms between case-control cohorts, unique genes identified by mRNA-seq from the ACCESS cohort were matched to corresponding Ensembl gene identifiers within the UCSF cohort to establish a uniform gene expression dataset prior to *WGCNA*. No subjects were considered outliers by complete-linkage agglomerative hierarchical clustering and network construction was carried out on 12,047 matched genes from all subjects after filtering for sparse gene expression (**Supplemental Figure S3A**). The Z-summary statistic, specifying the ACCESS cohort as the reference case-control data set, was utilized to assess module preservation between ACCESS and UCSF cohorts. Gene modules identified in the ACCESS co-expression network with Z-summary scores greater than 10 were deemed preserved between case-control cohorts and were considered for further analysis (46, 48).

Module eigengenes, obtained from specific gene expression of standardized modules between the ACCESS and UCSF cohorts, were extracted as representations of modular gene expression. Associations between gene modules and clinical features (gender, age, smoking history, and disease status) were evaluated by Spearman’s correlation and *p-values <*0.05 were considered significant. Additional Z-scored module eigengene expression associations were further assessed by Mann-Whitney U (MWU) test or linear regression models and considered significant if p-value < 0.05. Differential expression of genes between sarcoidosis cases and controls within modules significantly associated with disease in the ACCESS cohort was determined utilizing a moderated t-statistic at a predetermined false discovery rate (FDR) of 10% (*Benjamini-Hochberg adjusted* [*BH.adj*] *p-value <*0.1) (49). Subsequently, determination of biologic function of modules significantly associated with disease status and preserved between cohorts was performed utilizing the gene ontology of biologic processes from the Database for Annotation, Visualization and Integrated Discovery (DAVID Bioinformatics Database v6.8; https://david.ncifcrf.gov) (50, 51). Biologic processes identified within the modules found to have *BH.adj p-value <*0.1 to account for multiple testing were determined to be significantly enriched. The Human Protein Atlas (HPA) was utilized to determine whether specific PBMC subset enrichment was associated with gene expression within modules of interest (52, 53). Differences in PBMC subset enrichment were assessed within relevant modules utilizing Kruskal-Wallis (KW) one-way analysis of variance test followed by *post hoc* analysis using Dunn’s test with BH adjustment to account for multiplicity (p-values < 0.05 and < 0.1 were pre-specified as significant for KW and Dunn’s tests; respectively). Further details regarding network construction, module preservation, and cell subset enrichment are provided in the data supplement.

### Determination of master regulator transcription factors

To identify master regulator transcription factors capable of driving the changes in PBMC transcriptional programming (cell state transitions) from the healthy to the dysregulated immune response observed in sarcoidosis we applied the *Monster* algorithm (54). *Monster* infers master regulators capable of driving cell state transitions at the gene regulatory network level as described in the data supplement. In brief, it discriminates between transcription factors that alter or maintain unaltered targeting patterns between states. Regulators of cell state transitions are defined in the *Monster* algorithm as transcription factors that exhibit greater magnitude of change measured by differential transcription factor involvement (DTFI) within a gene regulatory network. To determine DTFI that underlie immune regulatory networks in health and disease, ACCESS cohort control samples (n=14) were defined as the baseline (reference) state and sarcoidosis samples (n=14) as the perturbed (final) state. DTFI significance was established if the false discovery rate (FDR) p-value based on the Monster Z-score method was <0.1.

### Subnetwork construction and analysis

Gene interaction subnetworks were derived from significant and preserved gene co-expression modules to explore the effect of aberrant transcriptional targeting on sarcoidosis related immune dysregulation defined by significant differences in cell subset proportions between cases and controls. To do so, state specific bipartite networks constructed by the *Monster* algorithm were extracted separately for the ACCESS cohort sarcoidosis cases and controls. Then bipartite networks were filtered to include significant DTFI and differentially expressed target genes with greater predicted edge weight in sarcoidosis compared to controls. Additional subnetwork reduction was performed to evaluate significantly enriched GO biologic processes associated with cellular proliferation or death assumed to alter PBMC subset proportions. Specifically, only target genes related to GO biologic processes involved in cell “aging/senescence,” “apoptosis,” “arrest,” “autophagy,” “cycle,” “death,” “differentiation,” “division,” “exhaustion,” “phagocytosis,” “proliferation,” and “quiescence” comprised the final subnetworks. Upon subnetwork construction, the Laplacian centrality measure for directed and weighted networks was utilized to perform topological analysis and determine transcription factor relevance (55, 56). Prior to bipartite network construction, PBMC subset proportions were defined by *in-silico* cellular deconvolution using *Cibersort* as described in the data supplement (57). Significant differences in cell proportions between sarcoidosis cases and controls within and between the ACCESS and UCSF cohorts were determined by MWU test p-values < 0.05.

To evaluate the relationship between target genes of the most central DTFI in individual subnetworks of interest and PBMC subset proportions, supervised classifier models with partial least squares discriminant analysis (PLS-DA) were constructed using data from the ACCESS cohort for model calibration and externally validated (58). To corroborate findings, two additional gene lists (n=50 genes per list), serving as negative controls, were derived at random from gene co-expression modules that were neither related to sarcoidosis nor preserved between the ACCESS and UCSF case-control cohorts. Initial classification with PLS-DA was performed only on significantly different *in-silico* imputed PBMC subsets in sarcoidosis cases from the ACCESS and UCSF cohorts after subjects were dichotomized into “high” and “low” groups based on corresponding median cell proportion values. Classification was based on thresholding of predicted response variables ≥0 or <0 in each cohort after cell proportions and target gene expression had been median centered and scaled to unit variance. PLS-DA model calibration on the ACCESS cohort was considered appropriate if the total number of misclassifications was less than 5 and the predictive accuracy was greater than 70% on the first principal component. PLS-DA validation model classification performance on the UCSF cohort was based on the area under the curve (AUC) of receiver operating characteristic (ROC) curves determined by the model’s ability to accurately predict “high” or “low” PBMC subset proportions in comparison to negative controls using Venkatraman’s test (59). Lastly, to substantiate findings, the predictive accuracy of target genes from PLS-DA models that were determined to be significant predictors of PBMC subsets of interest in the ACCESS and UCSF cohorts as well as the predictive accuracy of negative control models was further tested on *in-silico* imputed and clinical cell proportions from the UIC STAR cohort. PLS-DA validation model classification performance on *in-silico* imputed and clinical cell proportions for the UIC STAR cohort was based on AUC of ROC curves and Venkatraman’s test as performed on the UCSF cohort. Variable importance in projection (VIP) scores were calculated and utilized to determine the discriminatory power of each feature within PLS-DA models. Individual target genes with high VIP scores (>1) were considered the most relevant predictors of cell subset variability in sarcoidosis (58, 60).

## Results

### Subject characteristics

Our aim was to examine gene expression and transcriptional regulation patterns that might contribute to altered peripheral immunity in sarcoidosis. We obtained PBMCs from subjects with sarcoidosis within 6 months of diagnosis and their matched controls (n=14 per group) in the ACCESS cohort and performed mRNA-seq and network-based analyses. Baseline characteristics of the ACCESS cohort are outlined in **Table 1**. This cohort was predominantly female and relatively young with only a minority of cases (21.41%) above 50 years of age. Most reported a history of smoking but only 21.43% of cases and 28.57% of controls were active smokers at the time of study inclusion. The majority had definite pulmonary involvement; however, none were considered to have radiographic features consistent with Scadding stage 4 (i.e., pulmonary fibrosis) and median baseline spirometric values were within normal ranges for predicted values across the cases. Definite extrathoracic involvement was observed in 50% of cases and 64.3% had both definite pulmonary and extrathoracic involvement. Only two subjects were reported to have had erythema nodosum. None of the ACCESS cohort cases were reported to be on disease modifying anti-sarcoidosis medications at the time of inclusion and were considered to represent the baseline immunologic state for sarcoidosis.

**Table 1:** Baseline characteristics of sarcoidosis cases and matched controls in the ACCESS cohort based on responses or results reported on ACCESS Study data forms.

ACCESS cohort		Sarcoidosis cases n = 14 (50%)	Controls n = 14 (50%)	P-value
**Ethnicity (%)**	Caucasian	14 (100.0)	14 (100.0)	1
**Sex (%)**	Male	5 (35.7)	5 (35.7)	1
	Female	9 (64.3)	9 (64.3)	
**Age (%)**	<30 years	2 (14.3)	1 (7.1)	0.6583
	30-39 years	3 (21.4)	6 (42.9)	
	40-49 years	6 (42.9)	5 (35.7)	
	50-59 years	2 (14.3)	2 (14.3)	
	60-69 years	1 (7.1)	0 (0.0)	
**Smoking history (%)**	Never	6 (42.9)	5 (35.7)	0.6988
	Positive	8 (57.1)	9 (64.3)	
**Active smoker at time of inclusion (%)**	No	5	5	0.7715
	Yes	3	4	
**On treatment at time of inclusion (%)**	No	14 (100)	——	NA
	Yes	0 (0.0)	——	
**Scadding stage on chest radiograph**	0	3 (21.4)	——	NA
	1	6 (42.9)	——	
	2	3 (21.4)	——	
	3	2 (14.3)	——	
**Definite pulmonary involvement (%)**	No	2 (14.3)	——	NA
	Yes	12 (85.7)	——	
**Definite extra-thoracic involvement (%)**	No	7 (50.0)	——	NA
	Yes	7 (50.0)	——	
**Definite pulmonary and extra-thoracic involvement (%)**	No	5 (35.7)	——	NA
	Yes	9 (64.3)	——	
**Positive biopsy site**	Lung	5 (35.7)	——	NA
	Mediastinal/Hilar Lymph Node	6 (42.9)	——	
	Liver	1 (7.14)	——	
	Skin	1 (7.14)	——	
	Extrathoracic Lymph Node	1 (7.14)	——	
Baseline spirometry*	FEV1 (% predicted)	97.44 (13.80)	——	NA
	FVC (% predicted)	97.96 (15.51)	——	NA
	FEV1/FVC	77.46 (8.19)	——	NA

Definitive organ involvement refers to proof of features typical for sarcoidosis granulomas on biopsy (upon ruling out alternate causes of granulomas) or additional criteria based on the ACCESS study procedures manual, volume I **Table 3**-**1A** (https://biolincc.nhlbi.nih.gov/studies/access/). *Spirometry values shown as median and standard deviation. P-values are based on χ^2^-test for categorical variables. NA, not applicable.

A publicly available PBMC gene expression data set (GSE19314) was used to validate expression and transcriptional regulation analyses performed in the ACCESS cohort (**Table 2**) (29). A total of 47 Caucasian subjects, including 31 sarcoidosis cases and 16 controls, were identified and constituted the UCSF cohort. No differences were observed between cohorts with regards to sex when independently comparing cases or controls or upon comparison of all subjects between cohorts (χ^2^ test p-value >0.05). Significant differences were found with regards to age between cohorts. Specifically, UCSF cohort cases and controls were found to be significantly older than their ACCESS cohort counterparts (χ^2^ test p-value <0.05) with the majority of subjects in age groups 4 and 5 (40-49 and 50-59 years old; respectively). UCSF cohort sarcoidosis cases included 14/31 (45%) cases with low lung function defined by the study’s original parameters as forced expiratory volume in one second (FEV1) or forced vital capacity (FVC) percent predicted <80%. Individual subject data pertaining to other variables (smoking, Scadding stage, and treatment) was not publicly available. Nonetheless, in contrast to the ACCESS cohort, the original GSE19314 cohort included only 2/38 cases with a positive smoking history, 10/38 with Scadding stage 4 sarcoidosis, and at least 32% on systemic therapy for sarcoidosis.

**Table 2:** Baseline characteristics of sarcoidosis cases and controls from the “UCSF Sarcoidosis and Hypersensitivity Pneumonitis Cohort” (GSE19314) based on publicly available information in the gene expression omnibus (GEO) database.

UCSF cohort		Sarcoidosis casesn = 31 (66%)	Controls n = 16 (34%)	P-value
**Ethnicity (%)**	Caucasian	31 (100.0)	16 (100.0)	1
**Sex (%)**	Male	13 (41.9)	4 (25.0)	0.2522
	Female	18 (58.1)	12 (75.0)	
**Age (%)**	<30 years	0 (0)	0 (0)	0.8672
	30-39 years	4 (12.9)	1 (6.2)	
	40-49 years	6 (19.4)	4 (25.0)	
	50-59 years	12 (38.7)	7 (43.8)	
	60-69 years	9 (29.0)	4 (25.0)	
**Lung Function Group**	Low	14 (45.2)	——	NA
	High	17 (54.8)	——	

P-values are based on χ^2^-test for categorical variables.

NA, not applicable.

Unlike the Caucasian case-control cohorts, the UIC STAR cohort (n=30) consisted of a predominantly African American (66.7%) case series of subjects with sarcoidosis as detailed in the data supplement (**Table 3**). Within the UIC STAR cohort all subjects had evidence of pulmonary involvement of sarcoidosis. African Americans and Caucasians were comparable in terms of sex, age, smoking history, disease duration, disease severity, and treatment status. When compared to cases from the ACCESS and UCSF cohorts, no significant differences in sex or age were found (χ^2^ test p-value >0.05) but UIC STAR cohort subjects tended to be older than ACCESS counterparts (χ^2^ test p-value =0.071). Significant differences in smoking history were not observed between the UIC STAR and ACCESS cohorts (χ^2^ test p-value >0.05). Furthermore, pulmonary function between the UIC STAR and ACCESS cohorts was similar with only FEV1% predicted showing a trend towards significantly lower values in the UIC STAR cohort (median 86.04% *vs* 97.44%, MWU test p-value =0.095) despite the fact that the majority of subjects in the UIC STAR cohort had chronic disease (>3 years duration), 50% (15/30) had severe disease, and only 20% (6/30) were off of systemic therapy. Other differences between the UIC STAR cohort and UCSF cohort could not be assessed due to lack of publicly available data.

**Table 3:** Baseline characteristics of sarcoidosis cases from the “University of Illinois at Chicago Bernie Mac Sarcoidosis Translational Advanced Research Center (UIC STAR) Cohort”.

UIC STAR cohort		African Americans n = 20 (66.6%)	Caucasians n = 10 (33.3%)	P-value
**Sex (%)**	Male	9 (45.0)	5 (50.0)	0.7958
	Female	11 (55.0)	5 (50.0)	
**Age (%)**	30-39 years	3 (21.4)	0 (0.0)	0.3728
	40-49 years	5 (25.0)	4 (40.0)	
	50-59 years	9 (45.0)	3 (30.0)	
	60-69 years	3 (15.0)	3 (30.0)	
**Smoking history (%)**	Never	8 (40.0)	3 (30.0)	0.858
	Former	10 (50.0)	6 (60.0)	
	Current	2 (10.0)	1 (10.0)	
**On treatment at time of inclusion (%)**	No	5 (25.0)	1 (10.0)	0.3329
	Yes	15 (75.0)	9 (90.0)	
Approximate Time from Diagnosis*	Years	9.5 (8.44)	5 (6.72)	0.27
**Sarcoidosis Severity (%)**	Non-severe	9 (45.0)	6 (60.0)	0.699
	Severe	11 (55.0)	4 (40.0)	
Spirometry*	FEV1 (% predicted)	86.61 (23.25)	86.04 (23.72)	0.9999
	FVC (% predicted)	94.85 (23.51)	96.19 (22.31)	0.9828
	FEV1/FVC	74.72 (9.79)	73.91 (8.86)	0.4745
Total PBMCs*	kcells/µl	1.85 (0.79)	1.85 (0.67)	0.9223
Monocytes*	% of PBMCs	28.22 (10.28)	32.29 (11.82)	0.4541
CD4+ T-cells*	% of PBMCs	30.43 (10.36)	25.8 (11.86)	0.5588

*Continuous parameters shown as median and standard deviation. P-values are based on χ^2^-test for categorical variables and Mann-Whitney U-test for continuous variables.

### Dysregulation of cell cycle and apoptotic pathways is implicated in sarcoidosis

**Figure 1:**
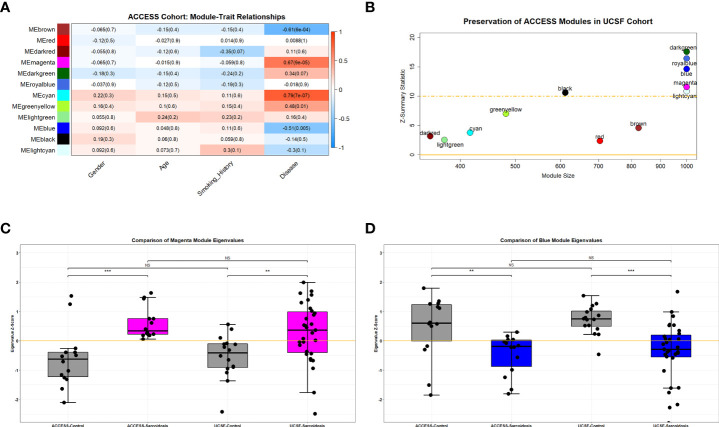
Preserved over-expressed and under-expressed modules in sarcoidosis. **(A)** Heatmap demonstrating WGCNA module-trait relationships in the ACCESS cohort. Columns correspond to clinical feature and rows correspond to module eigengenes. Cells depict the corresponding Spearman correlation coefficient and p-value (in parenthesis). Cell color indicates the strength of the correlation from strong negative (darker blue) to strong positive (darker red). Significance was determined by p-value <0.05. Cyan, greenyellow and magenta modules were found to be significantly correlated with disease (sarcoidosis), whereas blue and brown modules were found to be significantly but negatively correlated with disease. There were no significant relationships between module expression and gender, age, or smoking history. **(B)** Scatterplot demonstrating module size and module preservation between cohorts based on Z-summary statistic. The magenta and blue modules were strongly preserved (Z-summary statistic > 10) between the ACCESS and UCSF cohorts. **(C, D)** Module eigengenes, a measure of module expression, demonstrated that the magenta module was similarly over-expressed in sarcoidosis cases **(C)** and the blue module was similarly under-expressed in sarcoidosis **(D)** between cohorts. NS, non-significant; **p-value < 0.01; ***p-value < 0.001; Mann-Whitney U Test.

To analyze disrupted patterns of gene expression in sarcoidosis, mRNA-seq data obtained from the ACCESS cohort and microarray data from the UCSF cohort were filtered and normalized to identify 12,047 genes commonly expressed within both datasets. Utilizing *WGCNA*, 12 distinct modules of gene expression were identified within the 28 subjects sampled in the ACCESS cohort (**Supplemental Figure S3B** and **Supplemental Tables S2A, B**). Of the identified modules, 5 were significantly associated with disease status (*Spearman’s test p-value <0.05*) and one trended towards significance (*Spearman’s test p-value =0.07*) (**Figure 1A**). Three significant modules were positively correlated with sarcoidosis and two significant modules were inversely correlated with sarcoidosis. A total of 416, 481, and 1,379 genes were assigned to the significant and positively correlated modules cyan, greenyellow, and magenta, respectively. Gene expression in these modules was relatively increased in comparison to controls with 59 genes in the cyan module, 12 genes in the greenyellow module, and 287 genes in the magenta module determined to be significantly over-expressed in sarcoidosis (*BH.adj p-value <*0.1) (**Supplemental Figure S4A** and **Supplemental Table S3A**). In contrast, the significant modules inversely correlated with sarcoidosis included 57 of 1,331 genes in the blue module and 17 of 823 genes in the brown module that were significantly under-expressed (*BH.adj p-value <*0.1) (**Supplemental Figure S4A** and **Supplemental Table S3A**). Neither significant under-expression in positively correlated modules nor significant over-expression in inversely correlated modules was observed. No modules were significantly correlated with age or gender. The dark red module trended towards an association with smoking history (*Spearman’s test p-value <*0.1) but had no association with disease status. Thus, expression of unique genes within the modules significantly associated with disease was considered independent of age, gender, and smoking history.

To validate modules of interest, the modular framework derived from the ACCESS cohort was applied to the gene expression data from the UCSF cohort. Module preservation between case-control datasets was determined using the Z-summary statistic with scores greater than 10 indicating strong preservation (**Figure 1B**). Of the 5 modules significantly associated with disease status in the ACCESS cohort, only 2 modules were strongly preserved in the UCSF cohort: the magenta and blue modules. Compared to findings in the ACCESS cohort, the magenta module was similarly over-expressed and the blue module was similarly under-expressed in the UCSF cohort (**Figures 1C, D**). Even though there was similar modular expression in the UCSF cohort, the proportion of significantly over-expressed genes within the magenta module and under-expressed genes with the blue module was distinct (173/1,379 and 316/1,331 with *BH.adj p-value <*0.1; respectively) and likely attributable to clinical, demographic, and geographic heterogeneity between the cohorts (**Supplemental Figure S4B** and **Supplemental Table S3B**). Despite dissimilarities, consensus gene expression was ascertained (**Supplemental Figure S4C**) and 20.4% of significantly over-expressed genes in the magenta module as well as 8.4% of significantly under-expressed genes in the blue module were common to both cohorts (**Supplemental Figures S4D, E**). Many of the mutually significant genes, including BATF2, C1QB, CD1D, FCGR1A, ICAM1, IRF1, IL15RA, NOD2, SERPING1, SMARCD3, TYMP, and WARS have been associated with sarcoidosis in other bulk transcriptomic analyses of whole blood and PBMCs and are known to be involved in regulation of innate and adaptive immune responses and cell differentiation (25, 28, 30–32, 61–63). Importantly, member genes CD28, CD40LG, and LEF1 involved in T-cell activation, were noted to be under-expressed in both ACCESS and UCSF cohorts and this finding was consistent with a prior independent analysis of the ACCESS cohort (16).

**Figure 2:**
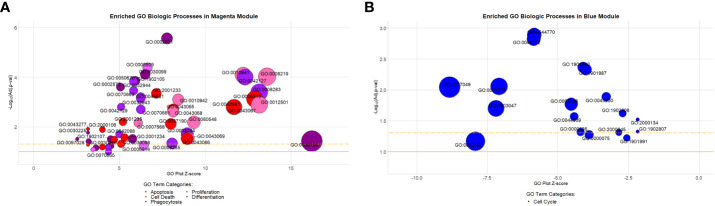
Gene ontology biologic processes enriched in the preserved magenta and blue modules. **(A)** Highlighted processes in the magenta module are associated with apoptosis, cell death, differentiation, phagocytosis, and proliferation. They comprised approximately 9% (53/587) of all significantly enriched processes within the module (Benjamini-Hochberg adjusted [BH-adj] p-value < 0.1). **(B)** Highlighted processes in the blue module are all associated with the cell cycle. They comprised approximately 24.7% (18/73) of all significantly enriched processes within the module (BH-adj p-value < 0.1). Description of GO terms are available in **Supplemental Tables S3A** (magenta) and **S3B** (blue). Bubble plot y-axis represents biologic process significance (-Log_10_ adjusted p-values) and x-axis represents the GO plot Z-score indicative of the likelihood that a biologic process is up (positive value) or downregulated (negative value). The gold solid horizontal line on the bubble plot represents a BH-adj p-value *=* 0.1 and the gold dashed horizontal line represents a BH-adj p-value *=* 0.05.

To define the molecular processes driving immune dysregulation in sarcoidosis, gene ontology analysis of the magenta and blue modules was performed using the DAVID pipeline (NIAID) (50, 51). The top 5 significantly enriched (*BH.adj p-value <*0.1) gene ontology terms that characterized the magenta module were consistent with fundamental notions of sarcoidosis and included: “immune system processes (GO:0002376),” “defense response (GO:0006952),” “immune response (GO:0006955),” “regulation of immune system processes (GO:0002682),” and “cell activation (GO:0001775)”. In contrast, the blue module was characterized by significant enrichment of “RNA metabolic processes (GO:0016070),” “nucleic acid metabolic processes (GO:0090304),” “gene expression (GO:0010467),” “nucleobase-containing compound metabolic processes (GO:0006139),” and “cellular aromatic compound metabolic processes (GO:0006725)” suggesting that sarcoidosis is not only marked by immune dysregulation but may also be a result of altered metabolic pathways. Other significantly enriched ontology terms related to multiple innate immunity and cellular immunity pathways and genes were also found in the magenta and blue modules, respectively (**Supplemental Tables S4A, B**). However, most relevantly, functional analysis also revealed significant enrichment of cell death related pathways in the magenta module and decreased expression of cell survival pathways in the blue module (**Figures 2A, B**).

**Figure 3:**
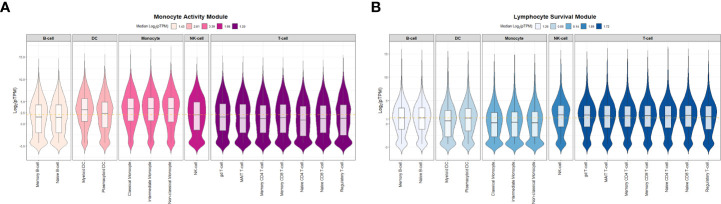
Absolute immune signal deconvolution of peripheral blood mononuclear cells based on the Human Protein Atlas. Violin plots demonstrate relative expression (log_2_-pTPM) of cell specific genes. **(A)** The Monocyte Activity Module (MAM, magenta) was characterized by high relative expression of monocyte and dendritic cell genes and low relative expression of lymphocyte genes. **(B)** The Lymphocyte Survival Module (LSM, blue) was characterized by high relative expression of lymphocyte genes and low relative expression of monocyte and dendritic cell genes. For **(A, B)** median log_2_-pTPM values per cell type (B-cells, Dendritic cells [DC], Monocytes, Natural Killer [NK] cells, and T-cells) are shown in the figure legends. Significant differences in relative expression of cell specific genes were identified between specific cell types within MAM and LSM (Kruskal-Wallis test p-value < 0.05). *Post hoc* analysis with Benjamini-Hochberg (BH) adjustment for multiple comparisons was performed with Dunn’s test and BH.adj p-value <0.1 was considered significant. In MAM, all *post hoc* comparisons assessing differences in relative expression of cell type specific genes were found to be significant with the exception of B-cell and T-cell genes which were comparable (BH.adj p-value =0.33). Similarly, in LSM all *post hoc* comparisons were significant except for NK-cell versus T-cells (BH.adj p-value =0.37). pTPM = normalized to transcripts per million protein coding genes. The gold dashed horizontal line represents the median log_2_-pTPM values among all genes in each module.

To determine if module-specific gene expression was driven by distinct immune cell types, absolute immune signal (ABIS) deconvolution was performed utilizing the Human Protein Atlas (HPA) and demonstrated that the magenta module was significantly associated with protein coding genes characteristic of monocytes and dendritic cells. The magenta module was therefore renamed the monocyte activity module (MAM) (**Figure 3A**). Conversely, the blue module was significantly associated with T-, B-, and NK lymphocyte protein coding genes, and was renamed the lymphocyte survival module (LSM) (**Figure 3B**) (52, 53). Taken together, these findings suggest that immune dysregulation leading to increased expression of MAM and decreased expression of LSM is likely to contribute to characteristic differential proportions of circulating cell subsets in sarcoidosis.

### Transcriptional states are linked to aberrant gene expression in sarcoidosis

In a prior gene co-expression analyses of peripheral blood and cutaneous lesions we noted that transcription factors, such as ETS1, IKZF3, LEF1, MYC, RORA, and SPI1 (PU.1), may be central players in aberrant processes associated with sarcoidosis (16, 43). In this study, in order to further examine the manner in which transcription factors contribute to dysregulated immune regulatory networks in sarcoidosis, we utilized *Monster* to independently infer master regulators driving cell state transcriptional programs and their target genes in MAM and LSM within the ACCESS cohort in a comprehensive and unbiased manner (54). The resultant analysis derived from gene expression data discriminated between transcription factors that maintain unchanged targeting patterns and those that experienced change within a gene regulatory network and defined differential transcription factor involvement (DTFI) based on the magnitude of targeting change. As *Monster* was performed to differentiate regulatory networks between cell states, transcription factors that only underwent small changes in targeting patterns between states were considered to fulfill “housekeeping” functions in both sarcoidosis cases and controls whereas those with significant changes in targeting patterns were deemed critical regulators of sarcoidosis.

**Figure 4:**
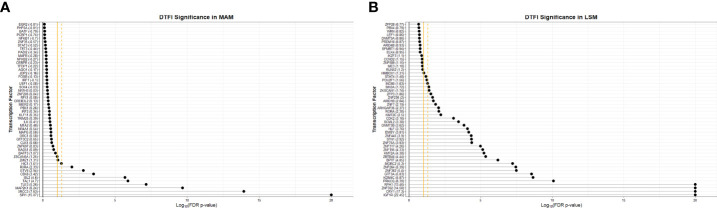
Significance of transcription factors based on differential transcription factor analysis. Dot plots demonstrate the significance of the top 50 transcription factors involved in the “transition” from health to sarcoidosis based on scaled differential transcription factor involvement (DTFI) within the MAM **(A)** and LSM **(B)** modules. DTFI was considered significant with a false discovery rate (FDR) p-value <0.1 utilizing the “z-score” method per the *Monster* algorithm. Gold solid vertical grid line represents a FDR p-value=0.1 and gold dashed vertical grid line represents FDR p-value=0.05. Numbers enclosed within parentheses next to transcription factor names on the y-axis represent scaled DTFI values standardized against the null distribution. Larger DTFI values indicate a greater magnitude of change in transcription factor gene targeting between states.

**Figure 5:**
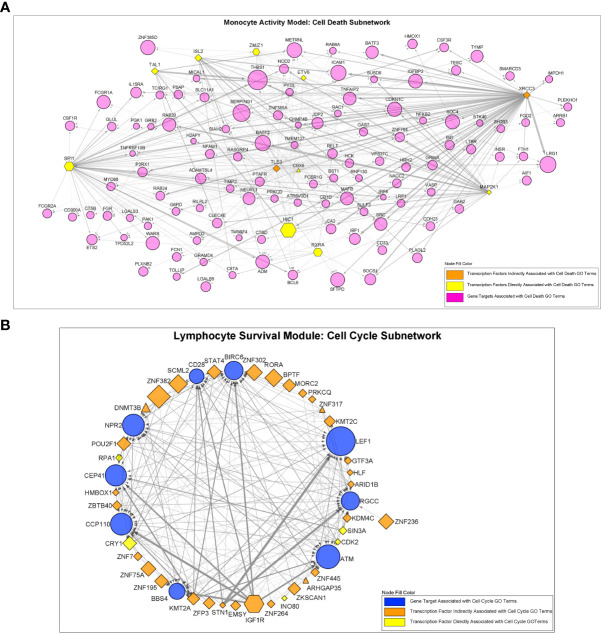
Regulatory interactions between transcription factors and target genes in the “Cell Death” and “Cell Cycle” subnetworks. **(A)** The MAM cell death subnetwork was comprised of a total of 123 nodes and 557 edges. Nodes consisted of 11 transcription factors with significantly altered regulatory activity and 112 target genes. **(B)** The LSM cell cycle subnetwork was comprised of a total of 45 nodes and 210 edges. Nodes consisted of 36 transcription factors with significantly altered regulatory activity and 9 target genes. For **(A, B)**, all transcription factors demonstrated significant differential transcription factor involvement (DTFI). Transcription factors are shown as hexagonal nodes if differentially over-expressed in MAM or differentially under-expressed in LSM and as rhomboid nodes if stably expressed (triangle shaped nodes in LSM represent 3 stably over-expressed transcription factors). Transcription factor color indicates direct or indirect relation with cell death or cell cycle GO terms. Target genes are depicted as magenta circles in **(A)** and blue circles in **(B)**. For all nodes in **(A, B)**, size is proportional to absolute log_2_-fold change based on differential expression analysis. Edges in **(A, B)** indicate an interaction between a transcription factor and target gene. Edge width represents the difference in weight of interaction between sarcoidosis cases and controls as determined by the *Monster* algorithm. MAM and LSM subnetwork structure is also depicted in **Supplemental Tables S5A, B**.

*Monster* analysis yielded a transcription factor to target gene network consisting of 106,029 edges in sarcoidosis cases and a corresponding network with differing strengths of interaction (edge weights) in controls from 81 transcription factors identified within 1,379 total genes in MAM. Examination of DTFI in MAM identified 11 transcription factors (13.58%) with significantly different targeting patterns in sarcoidosis compared to controls (FDR < 0.1) (**Figure 4A**). Analogous bipartite networks for sarcoidosis cases and controls in LSM produced networks with 133,120 edges and distinct edge weights. Within 1,331 total genes, 36/104 (34.62%) transcription factors were determined to have significant changes in targeting patterns (FDR < 0.1) (**Figure 4B**). Significant differences in gene expression resulting from altered transcription factor targeting in sarcoidosis were determined by constructing MAM and LSM subnetworks that encompassed only transcription factors with significant DTFI and differentially expressed target genes whose predicted strength of interaction in sarcoidosis was greater than in controls. Subsequently, to elucidate mechanisms leading to differential PBMC subset proportions in sarcoidosis, namely lymphopenia, subnetworks were further reduced. These transcriptional regulatory networks included transcription factors directly implicated with GO biologic processes significantly associated with cellular proliferation and death as well as those indirectly associated through targeting of genes directly involved in these processes. The resultant MAM “cell death” subnetwork (**Figure 5A** and **Supplemental Table S5A**) was comprised of 11 DTFI and 112 target genes (nodes) with a total of 557 interactions (edges), whereas the LSM “cell cycle” subnetwork (**Figure 5B** and **Supplemental Table S5B**) was comprised of 36 DTFI and 9 target genes with a total of 210 interactions.

**Figure 6:**
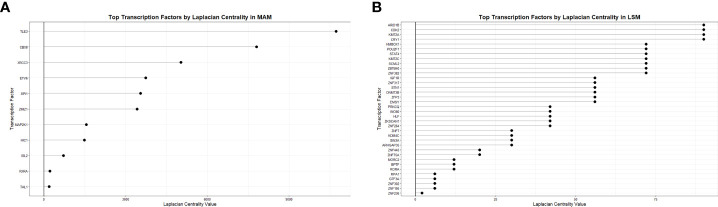
Transcription factor centrality in MAM Cell Death and LSM Cell Cycle subnetworks. Dot plots demonstrate significance of transcription factors regulating the MAM Cell Death **(A)** and LSM Cell Cycle **(B)** subnetworks based on Laplacian centrality in descending order.

To derive the most relevant transcription factors on subnetwork structure, Laplacian centrality was assessed. In the MAM cell death subnetwork, the 5 most central transcription factors were TLE3, CBX8, XRCC3, ETV6, and SPI1 (**Figure 6A**). These transcription factors had the most impactful regulatory effects on differential gene expression within the MAM subnetwork and are likely key regulatory features driving cell death through heightened innate immune activity. Similar analyses performed on the smaller LSM cell cycle subnetwork identified 4 equally relevant transcription factors: ARID1B, CDK2, KMT2A, and CRY1 responsible for driving expression changes associated with an altered lymphocytic cell cycle (**Figure 6B**). Collectively, altered activity by the transcription factors driving gene expression in the MAM and LSM subnetworks suggests that disrupted T-cell proliferation and activity in a pro-inflammatory background underlies peripheral immunity in sarcoidosis.

### Transcriptional state predicts differential proportions of PBMC subsets in sarcoidosis

**Figure 7:**
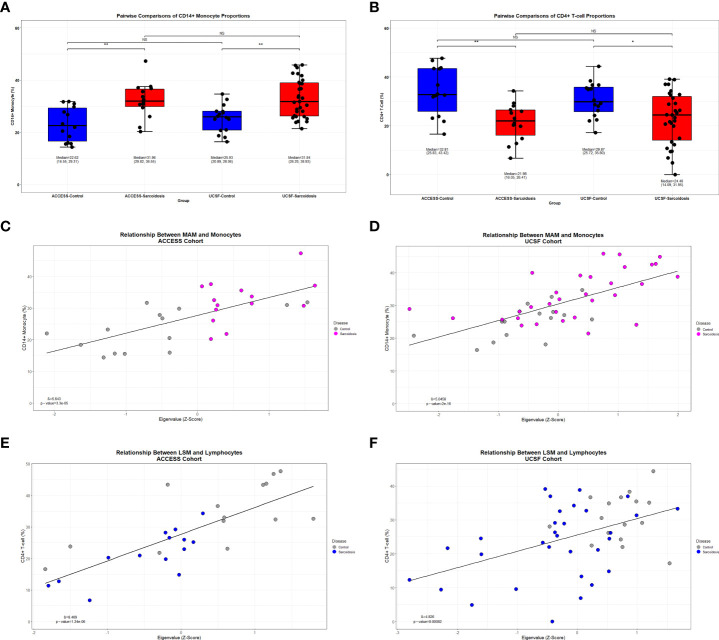
Decreased lymphocyte abundance in sarcoidosis linked to MAM and LSM transcription factors. **(A, B)** Box plots demonstrating the distribution of CD14+ monocyte and CD4+ T-cell in ACCESS and UCSF cohorts based on case-control grouping. PBMC subpopulation proportions were derived from *in-silico* cellular deconvolution utilizing *Cibersort* and the Immunostates gene signature matrix. Sarcoidosis cases in both cohorts were found to have significantly lower percentages of CD4+ T-cells when compared to controls within the same cohort. Comparable cell proportions were found among cases and controls between cohorts. For each group, corresponding median, 1^st^ quartile, and 3^rd^ quartile values are shown. P-values are shown above brackets for each pairwise comparison: NS, non-significant; *p-value < 0.05, **p-value < 0.01; Mann-Whitney U Test. **(C–F)** Linear regression identified significant associations between module eigengenes and cell subset proportions (p-value <0.05). CD14+ monocytes were significantly associated with MAM expression in the ACCESS **(C)** and UCSF **(D)** cohorts. CD4+ T-cells were significantly associated with LSM expression in the ACCESS **(E)** and UCSF **(F)** cohorts.

To test whether changes in transcriptional regulation were associated with significant differences in PBMC subset proportions, we first performed complementary *in-silico* cellular deconvolution analysis using *Cibersort* based on gene expression data from the ACCESS and UCSF cohorts (**Supplemental Table S6**) as previously performed in a pulmonary fibrosis cohort (57, 64). From queried PBMC subsets, only significantly increased CD14+ monocyte and significantly decreased CD4+ T-cell proportions were noted in sarcoidosis when compared against controls within both cohorts (MWU test p-value <0.05) (**Figures 7A, B**). Interestingly, despite cohort heterogeneity, CD14+ monocyte and CD4+ T-cell proportions in sarcoidosis cases and controls were comparable between cohorts. Moreover, linear regression analysis revealed that CD14+ monocytes were directly associated with MAM expression, and CD4+ T-cells were directly associated with LSM expression in both ACCESS and UCSF cohorts (**Figures 7C–F**). Thereby, these findings suggest that MAM and LSM are not only differentially expressed in the ACCESS and UCSF cohorts, but that their member genes are likely to modulate activity and characteristic compositional differences of circulating immune cells in sarcoidosis.

**Figure 8:**
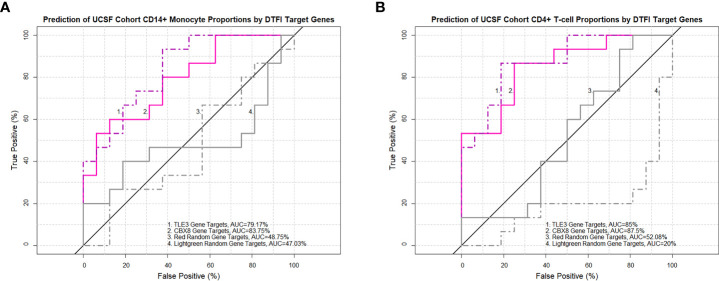
TLE3 and CBX8 target gene expression in MAM predicts imputed monocyte and lymphocyte proportions in the UCSF cohort. Receiver operating characteristic (ROC) curves demonstrate the area under the curve (AUC) as a measure of predictive performance for significant partial least squares discriminant analysis (PLS-DA) models in the validation cohort and negative controls derived from non-preserved modules (red and lightgreen) between ACCESS and UCSF cohorts. Predictive accuracy of CD14+ monocyte proportions **(A)** and CD4+ T-cell proportions **(B)** was significantly greater with TLE3 and CBX8 target genes in comparison to both negative control models (p-value < 0.05; Venkatraman test for AUC comparison with 10,000 bootstraps).

Next, to identify prominent features driving the relationship between transcription factors of interest and imputed PBMC subsets, PLS-DA models were constructed from MAM cell death and LSM cell cycle subnetworks. In addition, random gene lists from the red and lightgreen modules, determined to be the least preserved modules between the ACCESS and UCSF case-control cohorts based on the Z-summary statistic, were included as negative controls for the analysis. PLS-DA models were calibrated on the ACCESS cohort and first validated on the UCSF cohort. Subjects with CD4+ T-cells less than the corresponding median percentage in the ACCESS (21.98%) and in the UCSF (24.48%) cohorts defined the “low” lymphocyte groups. Conversely, CD14+ monocytes greater than or equal to 31.96% and 31.84% median percentages defined the “high” monocyte groups in the ACCESS and UCSF cohorts, respectively. Surprisingly, the LSM derived calibration model pertaining to transcription factors ARID1B, CDK2, CRY1, and KMT2A resulted in 5 misclassifications and <70% predictive accuracy for both cell subsets; while all other calibration models were capable of predicting subjects with low CD4+ T-cells and high CD14+ monocytes accurately and were considered for validation (**Supplemental Table S7A**). Validation on the UCSF cohort was performed on MAM derived PLS-DA models for the transcription factors CBX8, ETV6, SPI1, TLE3, and XRCC3 and classification performance was assessed by comparison of ROC curve AUC. Target genes of XRCC3 showed a trend towards significant lymphocyte classification and target genes of ETV6 were predictive of lymphocyte abundance. However, neither XRCC3 nor ETV6 target genes reliably determined CD14+ monocytes. Similarly, variable discriminatory capacity was observed with the SPI1 model which proved to be an accurate predictor of CD4+ T-cell abundance, but only exhibited a tendency towards significant prognostication of CD14+ monocytes (**Supplemental Table S7B**). In contrast, target genes of TLE3 and CBX8 were identified as significant predictors of both CD14+ monocyte and CD4+ T-cell abundance and significantly outperformed red and lightgreen random gene lists (**Figures 8A, B**).

**Figure 9:**
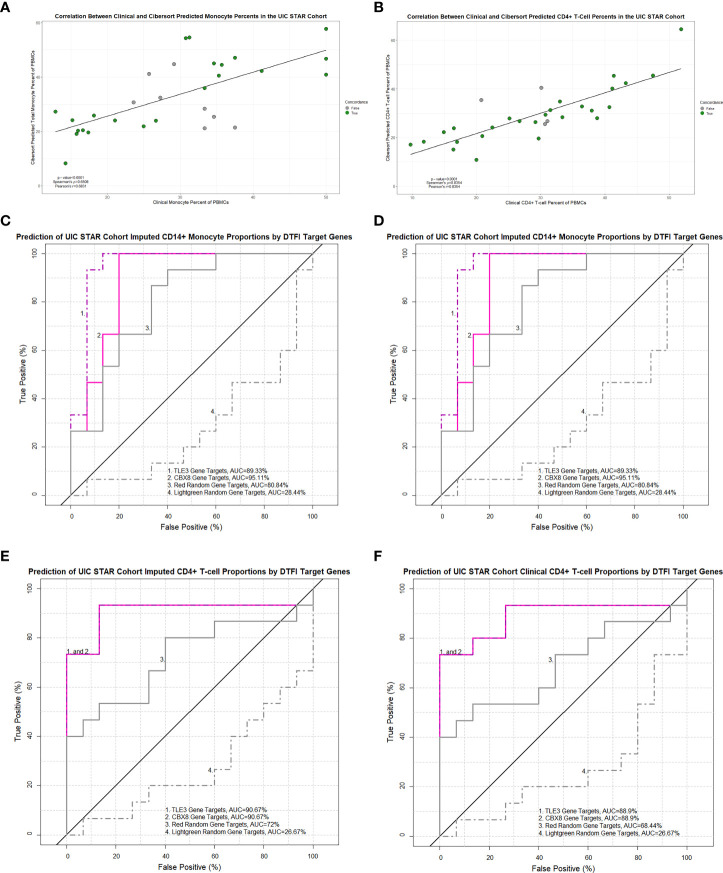
Imputed and clinical monocyte and lymphocyte proportions are strongly correlated in the UIC STAR cohort and predicted by TLE3 and CBX8 target gene expression in MAM. Significant correlations between imputed and clinical total monocyte percentage of PBMCs **(A)** and imputed and clinical CD4+ T-cell percentage of PBMCs **(B)** were identified (Pearson’s and Spearman’s test p-value <0.05). Concordance between imputed and clinical monocyte and lymphocyte “low” and “high” groups is depicted by colored circles. **(C–F)** Receiver operating characteristic (ROC) curves demonstrating the area under the curve (AUC) as a measure of predictive performance for significant partial least squares discriminant analysis (PLS-DA) models in the validation cohort and negative controls derived from non-preserved modules (red and lightgreen) between ACCESS and UIC STAR cohorts. Predictive accuracy of imputed CD14+ monocyte proportions **(C)** and total clinical monocyte proportions **(D)** was significantly greater with TLE3 and CBX8 target genes when compared to the lightgreen module random gene list (p-value < 0.05; Venkatraman test for AUC comparison with 10,000 bootstraps) but not the red module random gene list. Predictive accuracy of imputed CD4+ T-cell proportions **(E)** and clinical CD4+ T-cell proportions **(F)** was significantly greater with TLE3 and CBX8 target genes in comparison to the lightgreen and red module random gene lists (p-value < 0.05; Venkatraman test for AUC comparison with 10,000 bootstraps).

On account of superior performance, we sought to further validate and assess the general applicability of the ACCESS TLE3 and CBX8 PLS-DA calibration models on imputed and contemporaneous clinical cell proportions from the UIC STAR cohort. Prior to validation, the congruency between imputed and clinical total monocyte (as a surrogate for clinical enumeration of CD14+ monocytes by flow cytometry) and CD4+ T-cell proportions of PBMCs was ascertained. Imputed and clinical cell proportions in the UIC STAR cohort were highly and significantly correlated and demonstrated coefficients (**Figures 9A, B**) and root mean square error values (RMSE =6.41 and 10.15 for CD4+ T-cells and total monocytes, respectively) within the benchmarked ranges for *Cibersort* (57). Based on these findings, the corresponding median percentage of imputed CD14+ monocytes (16.84%) and total clinical monocytes (29.97%) as well as the corresponding median percentage of imputed (27.87%) and clinical (30.4%) CD4+ T-cells were utilized to designate subjects in the UIC STAR cohort into monocyte “high” and lymphocyte “low” groups. Concordance between imputed and clinical total monocyte (22/30) and imputed and clinical CD4+ T-cell (26/30) grouping was established (**Figures 9A, B**) and provides additional evidence to support the utilization of *in-silico* deconvolution to determine PBMC cell proportions in sarcoidosis. Moreover, median percentage values of imputed and clinical CD4+ T-cells for the UIC STAR cohort fell below the lower limit of normal established for CD4+ T-cell percent of total PBMCs (36.8%) in a large cohort of healthy adults that was inclusive of African American and Caucasian subjects and corroborated reduced CD4+ T-cell proportions in all subjects within the “low” group (65). Once congruency and presence of reduced CD4+ T-cell proportions were determined in the UIC STAR cohort, validation of the TLE3 and CBX8 PLS-DA models was performed. Analogous to the UCSF cohort validation, the predictive accuracy of TLE3 and CBX8 target genes was high with ROC curve AUC exceeding 85% while the predictive accuracy of the lightgreen module random gene list remained <50% in all UIC STAR cohort models. However, the predictive accuracy of the red random gene list was unexpectedly greater than what was observed in the UCSF cohort validation (>65% in the UIC STAR cohort models versus ~50% in the UCSF cohort models) (**Figures 9C–F** and **Supplemental Tables S7C, D**). Comparison of ROC curve AUC did not demonstrate greater classification of monocytes between the TLE3 or CBX8 target genes and the red random module gene list (Venkatraman test p-value >0.05). Nonetheless, both models demonstrated significantly greater monocyte classification performance when compared to the lightgreen random module gene list (Venkatraman test p-value <0.05) which suggests that in addition to TLE3 and CBX8 target genes, genes within in the red module, are likely to be implicated in modulation of monocyte activity in the UIC STAR cohort. Notwithstanding, target genes of TLE3 and CBX8 significantly outperformed the red and lightgreen random gene lists and accurately predicted imputed and clinical CD4+ T-cell abundance in the UIC STAR cohort (Venkatraman test p-value <0.05) further substantiating the association between altered transcription factor targeting and differential PBMC proportions in sarcoidosis.

The TLE3 and CBX8 PLS-DA models were further parsed to identify target genes in the MAM cell death subnetwork most likely associated with the characteristic imbalance of PBMC proportions observed in sarcoidosis. From 102 target genes of TLE3, 46 genes were found to have significantly high VIP scores (>1) in the CD4+ T-cell model and 37 in the CD14+/total monocyte model. Similarly, 40/87 and 34/87 target genes had high VIP scores in the corresponding CBX8 models. These genes represented the most influential determinants of PBMC proportions and likely comprise a core gene signature of sarcoidosis immune dysregulation (**Supplemental Table S7E**). Among the highly influential target genes identified between the ACCESS and UCSF cohorts, several were significantly over-expressed in both cohorts despite heterogeneity between them. As a result, consensus differentially expressed target genes, FCGR1A, IL15RA, LGALS9, P2RX1, RAB24, SERPING1, SMARCD3, and TYMP were considered key determinants of CD4+ T-cell proportions in the UCSF cohort, whereas BATF2, CD1D, FCGR1A, ICAM1, MICAL1, NOD2, RELT, and TYMP were considered key determinants of CD14+ monocyte proportions in the UCSF cohort. Notably, LGALS9, MICAL1, P2RX1, RAB24 and RELT, were not identified within the previously referenced whole blood or PBMC gene signatures (25, 28, 30–32, 61–63). Accordingly, consensus differentially expressed highly influential target genes between the ACCESS and UIC STAR cohorts were considered key determinants of CD4+ T-cell proportions and monocyte proportions in the UIC STAR cohort. In comparison to key determinants in the UCSF cohort, the majority of key determinants of cell proportions for the UIC STAR cohort were distinct targets of TLE3 and CBX8. In total, 23 genes were considered key determinants of CD4+ T-cells, among which ATP6V0D1, CD300A, FCGR1A, G6PD, NACC2, NFAM1, RAB24, SPI1, TM9SF4, and VASP were the top predictors with VIP scores >1.25. Key determinants of monocytes included ADM, BCL6, FCER1G, FCGR1A, FTH1, HCK, HRH2, LGALS3, NEURL1, NFAM1, and PTAFR. With the exception of RAC1 in the CD4+ T-cell model, BCL6 in the monocyte model, and FCGR1A and PTAFR in both models, all other key determinant genes for the UIC STAR cohort were not identified within previously referenced whole blood or PBMC gene signatures (25, 28, 30–32, 61–63). However, some UIC STAR cohort key determinant genes, such as ATP6V0D1, FTH1, G6PD, HCK, and SPI1 have been previously associated with sarcoidosis in other studies (43, 66, 67). Interestingly, among key determinants of lymphocyte and monocyte models, only FCGR1A was identified as being differentially overexpressed across all three cohorts. Altogether, this provides evidence that over-expression of specific gene targets of TLE3 and CBX8 involved in processes related to cell death and proliferation in MAM may exert a suppressive function towards CD4+ T-cells while inciting CD14+ monocytes through various mechanisms that contribute to characteristic peripheral lymphopenia and monocyte expansion in sarcoidosis.

## Discussion

Aberrant systemic T-cell activity is crucial in the immunopathogenesis of sarcoidosis and peripheral lymphopenia is of clinical significance (4, 6, 7, 13–15, 68, 69). Specifically, we have previously noted that decreased peripheral lymphocyte counts are related to increased FDG-avidity on PET scan and sarcoidosis severity (4, 7). Furthermore, our prior transcriptomic analyses of PBMCs uncovered that aberrant micro-RNA and gene expression patterns in sarcoidosis are associated with reductions in peripheral lymphocyte counts and pulmonary function decline (1, 16). However, despite the apparent interdependence between lymphopenia and sarcoidosis activity, regulatory mechanisms driving functional changes in lymphocytes have yet to be fully elucidated. In this study, our systematic strategy facilitated determination of master regulator transcription factors associated with lymphopenia and other features of immune dysregulation in sarcoidosis not readily identifiable by conventional differential expression analyses. We identified an over-expressed innate immunity module predominantly driven by monocytes (MAM) associated with cell death and an under-expressed module driven by lymphocytes (LSM) associated with cell cycle between sarcoidosis cases and matched controls in the ACCESS cohort. These modules were found to be preserved in the UCSF cohort even though significant heterogeneity existed between cohorts. Gene expression changes in MAM and LSM were primarily related to differential transcription factor involvement. In LSM, changes driven by ARID1B, CDK2, KMT2A, and CRY1 surprisingly did not predict CD4+ T-cell abundance. Contrastingly, altered regulatory activity of transcription factors TLE3, CBX8, ETV6, and SPI1 in MAM proved to be significantly associated with reduced CD4+ T-cell abundance and concurrent altered regulatory activity of TLE3 and CBX8 was significantly related with increased CD14+ monocyte abundance in the ACCESS and UCSF cohorts. Our significant findings in MAM, suggesting that altered targeting activity of TLE3 and CBX8 plays a role in determining peripheral lymphocyte reduction and monocyte expansion, were further substantiated on imputed and clinical cell proportions in the UIC STAR cohort. Overall, our results indicate that disrupted T-cell proliferation and activity in a pro-inflammatory background underlies peripheral lymphopenia in sarcoidosis and highlights the link between innate and adaptive immune dysregulation in the pathogenesis of sarcoidosis.

By analyzing gene expression regulation, we sought to discern disrupted regulatory pathways driving changes in lymphocyte activity and survival. Traditionally proposed mechanisms of peripheral lymphopenia uphold that it is a consequence of T-cell sequestration, infiltrated bone marrow, or impaired survival and function of regulatory T-cells (4, 9–11, 13, 14). However, our data suggests that peripheral lymphopenia may also depend on altered transcription factor targeting and dysregulation of cellular interplay within the circulating monocyte-lymphocyte axis. Interestingly, this is in line with a single-cell transcriptomic analysis of PBMCs from sarcoidosis cases and controls which concluded that sarcoidosis immune dysregulation may involve convergence of distinct arms of the innate and adaptive immune response (33). Additionally, our analysis of the ACCESS cohort argues that dysregulation of processes responsible for lymphocyte survival may be experienced early in the course of the disease in treatment naïve individuals and contests the notion that longstanding antigenic exposure is a prerequisite for T-cell exhaustion and peripheral lymphopenia (8, 68).

We characterized two significant and preserved gene co-expression modules of sarcoidosis and the transcription factors that influence immune network dysregulation related to PBMC function and survival. The best predictor of low CD4+ T-cell abundance was not LSM, which included lymphocyte activity genes, such as CD28, CD40LG, and LEF1, necessary for T-cell effector function, but MAM. Within MAM, aberrancies inciting transcriptional up-regulation of monocyte activity and cell death related processes may be instrumental to the development of paradoxical peripheral lymphopenia. Specifically, significantly altered regulatory activity of transcription factors CBX8, ETV6, SPI1, TLE3, and XRCC3 was central to the cell death subnetwork. The gene targets of ETV6, SPI1, and XRCC3 did not satisfy our criteria to consider these transcription factors significantly impactful as master regulators on circulating lymphocyte and monocyte proportions. However, it is worth noting that these transcription factors have established roles in DNA damage repair, cell proliferation, cell differentiation, and TNF and interferon inflammatory pathways in malignancies and infections previously associated with sarcoidosis (26, 70–80). Furthermore, SPI1, which was significantly over-expressed in the ACCESS cohort and demonstrated the greatest magnitude of transcriptional targeting change in MAM, was identified in our prior work as a hub gene in a sarcoidosis co-expression network (43). Thus, it is still possible that these transcription factors and their target genes lead to aberrancies of other transcriptional programs associated with sarcoidosis immune dysregulation within the global MAM network in monocytes, and possibly in lymphocytes, and require further assessment.

CBX8 and TLE3 were determined to be master regulators within the MAM cell death subnetwork. Unlike the target genes of XRCC3, ETV6, and SPI1, the target genes of CBX8 and TLE3 were predictive of CD4+ T-cell and CD14+ monocyte proportions in the UCSF cohort and their predictive ability was substantiated on the UIC STAR cohort. CBX8, a polycomb protein, is involved in DNA damage repair and has been linked to cell proliferation and differentiation through epigenetic modifications (81, 82). Interestingly, polycomb proteins can serve as tumor suppressors or promoters capable of inducing changes in cellular transcriptional programming that result in altered survival and proliferation of hematologic progenitors in various malignancies (83). Functional changes of CBX8 may also alter its role as a transcriptional repressor and cause dysregulation of mechanisms related to differentiation and exhaustion of hematologic progenitors (81). Therefore, aberrant CBX8 regulatory activity may contribute to the dysregulated immune response observed in sarcoidosis as a result of epigenetic modifications of immune cell progenitors in addition to functional changes within PBMCs. Distinctively, TLE3 is thought to modulate immune function by upregulating PPAR activity which interferes with transcription complexes such as STAT1 and inhibits Th1 pro-inflammatory cytokines leading to suppressed inflammatory gene activation (84, 85). Contrastingly, PPAR-γ is deficient in alveolar macrophages of those with chronic active sarcoidosis and both PPAR-α and PPAR-γ are significantly under-expressed in peripheral blood and bronchoalveolar lavage fluid derived CD4+ T-cells of those with the non-Löfgren phenotype, likely contributing to the intense Th1 responses observed in the disease (84, 86). Additionally, in conjunction with TCF/LEF family transcription factors, members of the TLE family of co-repressors antagonize canonical WNT signaling and activation and influence inflammatory and anti-microbial responses (87–89). However, bronchoalveolar lavage fluid cells in sarcoidosis exhibit increased activation of the WNT signaling pathway (90). Furthermore, WNT signaling pathway dysregulation in sarcoidosis has been proposed as a consequence of regulatory targeting by differentially expressed micro-RNAs in PBMCs (91). Notably, we previously identified TLE3 among the predicted targets of hsa-miR-22-5p, hsa-miR-30e-3p, and hsa-miR-4306 within a diagnostic and prognostic micro-RNA signature associated with sarcoidosis immune dysregulation (1, 91). Aberrant post-transcriptional regulation may instigate altered transcriptional targeting of TLE3 and impede its typical antagonistic immunomodulatory functions. Consequently, signaling through unchecked PPAR or WNT pathways may provoke uninhibited activation of pathways associated with immune dysregulation in sarcoidosis. Taken together, over-expression of MAM and the dysregulated innate immune processes included within likely favor a chronic maladaptive inflammatory state in sarcoidosis by promoting lymphocyte dysfunction and loss of effector functions (92, 93). Selectively targeting and modulating CBX8 and TLE3 activity may represent a promising novel strategy for management of sarcoidosis.

Our findings in the MAM cell death subnetwork indicate that immune cell imbalances are significantly related to an aberrantly expressed core gene signature modulated by altered CBX8 and TLE3 regulatory activity. Within this core signature, preserved differential over-expression was found between the ACCESS cohort and 14 genes in the UCSF cohort and between the ACCESS cohort and 31 genes in the UIC STAR cohort. These genes exhibited high discriminatory capacity and were considered the most influential predictors and key determinants of CD14+/total monocyte expansion and CD4+ T-cell depletion. Despite accurate prediction of cell proportions in both the UCSF and UIC STAR cohorts, little overlap was observed between key determinants. This finding was not surprising given the variability that is observed among differential expression gene signatures in sarcoidosis and the fact that our predictive models were calibrated on treatment naïve subjects early in the course of their disease from the ACCESS cohort and validated on demographically and clinically heterogenous cohorts (1, 25, 28–34). Nevertheless, among key determinant genes in both UCSF and UIC STAR cohorts we identified genes that provide insight into underexplored mechanisms that may account for lymphopenia in sarcoidosis, such as the galectins, LGALS-3 and LGALS-9, which serve as both drivers of T-cell death and perpetuators of T-cell exhaustion (94–99). However, the plausibility of key determinant genes to function as modulators of sarcoidosis related immune dysregulation and peripheral lymphopenia is their involvement in previously described processes implicated with disease immunopathogenesis. In this regard, UCSF cohort related genes BATF2, SMARCD3, and SERPING1 are known to modulate the PD-1/PD-Ls pathway, the PPAR pathway, and the complement system, respectively (15, 68, 69, 84, 86, 100, 101). Similarly, UIC STAR cohort related genes such as RAC1 and ATP6V0D1 have been previously proposed to induce dysfunctional autophagy-related pathways in sarcoidosis and RAC1 and others such as CD300A, CSF3R, G6PD function as drivers of monocyte differentiation or as modulators of the innate immune response and promoters of inflammatory cytokines. SPI1, the TF with the greatest DTFI in MAM, was found to be a key determinant of CD4+ T-cells in the UIC STAR cohort and has specifically been proposed as an effector of the innate immune response that interacts with multiple genes previously associated with sarcoidosis including the STAT family transcription factors, FCγ receptors, and Toll receptors (66, 67, 79, 102–110). Additionally, underscoring the concept that lymphocyte dysfunction in sarcoidosis results, at least in part, from crosstalk with hyperactive monocytes, we found that key determinants of the UCSF and UIC STAR cohorts fulfill biologic functions related to regulation of vesicle-mediated transport, regulation of cellular secretion, cell signaling, cell communication, and lymphocyte mediated immunity and activation. Most intriguingly, we identified several aberrantly expressed key determinant genes including FCGR1A, ICAM1, IL15RA, and NOD2 in the UCSF cohort and CD300A, FCGR1A, G6PD, FCER1G, RAC1, SPI1 that have been previously described in sarcoidosis and have the potential to directly or indirectly dysregulate the TNF pathway (32, 66, 67, 79, 102–117). Notably, the TNF pathway is a key modulator of cell-cell interactions and cell death that plays a major role in formation and maintenance of granulomas. Moreover, enhanced TNF-α expression has also been linked to CD14+ monocytes in non-Löfgren and pulmonary monocytes in progressive phenotypes of sarcoidosis (80, 118, 119). Among key determinant genes related to the TNF pathway, FCGR1A was determined to be differentially over-expressed at relatively high levels all three cohorts and was established as a significant predictor of both CD14+ monocytes and CD4+ T-cells. Interestingly, Fc-γ receptors coordinate innate and adaptive cellular crosstalk through interactions with Toll-like receptors and evidence supports their use as biomarkers in sarcoidosis as well as various malignancies and active tuberculosis. This suggests that FCGR1A could serve as therapeutic target to mitigate TNF mediated immune dysregulation in sarcoidosis (103, 104, 120–123). Collectively, our findings suggest that master regulator transcription factors likely promulgate processes related to immune dysregulation in sarcoidosis through transcriptional programs that execute similar functions *via* different mechanisms. Consequently, aberrant expression of distinct key determinant genes related to specific phenotypes adversely impact monocyte-lymphocyte interaction pathways and contribute to maladaptive peripheral inflammatory signaling which in turn is associated with development of lymphopenia and sarcoidosis progression.

In this study, we leveraged gene expression data from various high-throughput “-omic” platforms and clinically heterogenous cohorts to determine an immune regulatory network and gene signature associated with lymphopenia in sarcoidosis and provide a methodology to incorporate data from multiple studies to improve our understanding of the immunopathogenesis of sarcoidosis. Even though there are advantages to utilizing a well-defined, matched, and treatment naïve case-control cohort of sarcoidosis with mixed phenotypes, we recognize that there are several limitations to our study that will need to be addressed in the future. As noted, by utilizing the ACCESS cohort as a reference data set, our study was possibly limited in its ability to identify pertinent findings that may be held in other gene modules such as the darkred module that demonstrated a trend towards an association with smoking and the red module that may represent a distinctly preserved signature of immune dysregulation in sarcoidosis that is influenced by differences in race, treatment, or other features that contributed to cohort heterogeneity. A larger study utilizing a different analytical approach or different parameters to contrast specific sarcoidosis phenotypes with other granulomatous and lymphopenic diseases, in addition to health, might have been better able to target transcription factors and pathways of interest in sarcoidosis and increase the statistical power and specificity of our findings. Additionally, though our findings on the UIC STAR cohort suggest that our gene signature is applicable to African Americans with sarcoidosis, our findings are likely to be more representative of immune dysregulation in Caucasians with sarcoidosis. It is crucial that subsequent studies focus on African Americans to better elucidate specific mechanisms underlying disease pathogenesis unique to this population and equitably address disparities. Furthermore, this study utilized data obtained from different “bulk” transcriptomic methodologies and demonstrated preservation of findings; however, it should also be noted that despite efforts to rigorously normalize data and utilize statistics that are agnostic to type of “-omic” platform utilized (such as the Z-summary statistic and PLS-DA models) the possibility that gene selection bias was introduced into our signature by a specific platform cannot be fully discounted. By utilizing “bulk” transcriptomic methodologies our study was also limited in its ability to directly assess involvement of specific PBMC subsets. To address this, in-depth single-cell transcriptomic analyses utilizing the same methodology could be employed to provide a comprehensive assessment of cell-specific dysregulation. Our analytical strategy also relied on *in-silico* methodologies to determine transcription factor involvement and impute immune cell populations and despite the congruency observed on cell proportions from clinical tests in the UIC STAR cohort *in-vitro* or *in-vivo* validation will still be required in future studies to further substantiate these findings. With respect to the *in-silico* methods, *Monster* and *Cibersort*, we note that both make use of input datasets to derive associations with transcription factors and cell types, and neither dataset was developed on sarcoidosis patients. Lastly, other limitations arise from the depth and accuracy of information stored in the databases that were utilized to generate the input datasets and determine functional annotation which can be addressed by re-analyzing the data in the future as information within public databases is expanded with information derived from more advanced technologies.

In conclusion, we determined master regulator transcription factors and target genes of immune dysregulation that are linked to characteristic paradoxical lymphopenia in sarcoidosis. Notably, we identified that differential transcriptional activity in monocytes was the best predictor of low lymphocyte counts. Crosstalk between monocytes and lymphocytes possibly involving the TNF pathway and Fc-γ receptors, as well as other features of innate immunity were the most likely dysregulated pathways involved with lymphopenia. Previous studies have established roles for both monocyte hyperactivity and T-lymphocyte dysfunction in sarcoidosis pathogenesis, but those analyses were limited in their assessment of the functional relationship between monocytes and lymphocytes (13, 33, 68, 124). As there is a continued need to evaluate the causes of immune dysregulation and discover targeted therapies to mitigate progression, the relationship between lymphocytes and monocytes, and how their interactions shape sarcoidosis outcomes, warrants further investigation.

## Data availability statement

The datasets presented in this study can be found in online repositories and the Supplementary Material. The names of the repository/repositories and accession numbers can be found in the article/**Supplementary Material**. Additional data requests can be made by contacting the corresponding author.

## Ethics statement

This study was reviewed and approved by the University of Illinois Institutional Review Board (Protocol #s: 2019-0452 and 2016-0063). Written and informed consent was not required for protocol 2019-0452 in accordance with the national legislation and the institutional requirements. Written and informed consent was obtained from all subjects who participated in protocol 2016-0063 in accordance with the national legislation and the institutional requirements.

## ACCESS research group

A Case Control Etiologic Study of Sarcoidosis (ACCESS) Research Group Clinical Centers:

*Beth Israel Deaconess Medical Center*: Steven E. Weinberger, M.D.; Patricia Finn, M.D.; Erik Garpestad, M.D.; Allison Moran, R.N.

*Georgetown University Medical Center*: Henry Yeager, Jr., M.D.; David L. Rabin, M.D.; Susan Stein, M.A.

*Case Western Reserve University - Henry Ford Health Sciences Center*: Michael C. Iannuzzi, M.D.; Benjamin A. Rybicki, Ph.D.; Marcie Major, R.N.; Mary Maliarik, Ph.D.; John Popovich, Jr., M.D.

*Johns Hopkins University School of Medicine*: David R. Moller, M.D.; Carol J. Johns, M.D.*; Cynthia Rand, Ph.D.; Joanne Steimel, R.N.

*Medical University of South Carolina*: Marc A. Judson, M.D.; Susan D’Alessandro, R.N., Nancy Heister, R.N.; Theresa Johnson, R.N.; Daniel T. Lackland, Dr.P.H.; Janardan Pandey, Ph.D.; Steven Sahn, M.D.; Charlie Strange, M.D.

*Mount Sinai Medical Center*: Alvin S. Teirstein, M.D.; Louis DePalo, M.D.; Sheldon Brown, M.D.; Marvin Lesser, M.D.; Maria L. Padilla, M.D.; Marilyn Marshall

*National Jewish Medical and Research Center*: Lee S. Newman, M.D., M.A.; Cecile Rose,M.D., M.P.H.; Juliana Barnard, M.A.

*University of Cincinnati Medical Center*: Robert P. Baughman, M.D.; Elyse E. Lower, M.D.; Donna B. Winget

*University of Iowa College of Medicine*: Geoffrey McLennan, M.D., Ph.D.; Gary Hunninghake, M.D.; Chuck Dayton, B.S.Pharm.; Linda Powers, M.S.

*University of Pennsylvania and Medical College of Pennsylvania - Hahnemann*

*Medical Centers*: Milton D. Rossman, M.D.; Eddy A. Bresnitz, M.D.; Ronald Daniele, M.D., Jackie Regovich, M.P.H.; William Sexauer, M.D.

*National Heart, Lung, and Blood Institute*: Robert Musson, Ph.D.; Joanne Deshler; Paul Sorlie, Ph.D.; Margaret Wu, Ph.D.

*Study Chairman*: Reuben Cherniack, M.D.

*Study Co-Chairman*: Lee Newman, M.D.

*Clinical Coordinating Center*

Clinical Trials & Surveys Corp.: Genell L. Knatterud, Ph.D., Michael L. Terrin, M.D., Bruce W. Thompson, Ph.D., Kathleen Brown, Ph.D., Margaret Frederick, Ph.D., Frances LoPresti, M.S., Patricia Wilkins, B.S., Martha Canner, M.S., Judy Dotson

*Central Repository:*

McKesson Bioservices (September, 1996 to November, 1998): Steve Lindenfelser BBI-Biotech Research Laboratories (December, 1988 to present): Mark Cosentino, Ph.D.* Deceased.

*Central Laboratories:*

DNA Core Laboratory: Mary Maliarik, Ph.D.

BAL Central Laboratory: Robert Baughman, M.D.

HLA Class II Typing Laboratory: Milton Rossman, M.D.; Dimitri Monos, Ph.D.; Chung Wha Lee, Ph.D., Boyana Cizman, Ph.D.

Etiologic Antigen in Kveim Reagent Laboratory: David Moller, M.D.

Immunogenetics Laboratory: Janardan Pandey, Ph.D.

L-Forms Core Laboratory: Peter Almenoff, M.D.; Ian Brett, Sheldon Brown, M.D.; Marvin Lesser, M.D.

Pathogenic T Cells Laboratory: Lee Newman, M.D.; Brian Kotzin, M.D.

Ribosomal DNA Core Laboratory: Geoffrey McLennan, M.D., Ph.D., Gary Hunninghake, M.D.

RNA Core Laboratory: Patricia Finn, M.D.

Random Digit Dialing Interview Group:

Telesurveys Research Associates: Richard D. Jaffe, M.A.

*Executive Committee:*

Reuben Cherniack, M.D. (Chair)

Robert P. Baughman, M.D. (9/1/98-8/31/99)

Joanne Deshler

Michael C. Iannuzzi, M.D.; (9/1/96-8/31/97; 9/1/00-6/30/01)

Marc A. Judson, M.D. (9/1/96-8/31/97); 9/1/00-6/30/01)

Genell L. Knatterud, Ph.D.

Geoffrey McLennan, M.D. (9/1/97-8/31/98)

David R. Moller, M.D. (9/1/95-3/31/96; 9/1/99-8/31/00)

Robert A Musson, Ph.D.

Lee S. Newman, M.D.

Milton D. Rossman, M.D. (8/1/95-3/31/86; 9/1/99-8/31/00)

Alvin S. Teirstein, M.D. (9/1/97-8/31/98)

Michael L. Terrin, M.D. M.P.H.

Steven E. Weinberger, M.D. (9/1/97-3/31/98)

Henry Yeager, Jr., M.D. (9/1/98-8/31/99)

Data Safety and Monitoring Board:

William Martin, M.D. (Chair)

Takamaru Ashikaga, Ph.D.

David B. Coultas, M.D.

Gerald S. Davis, M.D.

Fred Gifford, Ph.D.

James J. Schlesselman, Ph.D.

Diane Stover, M.D.

*Ex Officio:*

Reuben Cherniack, M.D., Genell L. Knatterud, Ph.D., Robert Musson, Ph.D.

Lee Newman, M.D.

## Author contributions

CA, YH, CS and BT conceived, designed experiments, and performed experiments. Specimens and clinical data were collected by the ACCESS Research Group. CA, YH, and NE performed ACCESS clinical database abstraction. WW indexed, annotated and quantified transcriptomic libraries. CA, CAS, YH and BT analyzed data. DP, PF contributed reagents and materials. CA, CAS, YH, NS, DP and PF wrote the manuscript. All authors contributed to the article and approved the submitted version.

## Funding

Support for this study was provided by NIH grant numbers R01 HL138628-01A1S1, T32 HL082547, and F30 HL137267 for the University of Illinois at Chicago Division of Pulmonary, Critical Care, Sleep and Allergy.

## Conflict of interest

The authors declare that the research was conducted in the absence of any commercial or financial relationships that could be construed as a potential conflict of interest.

## Publisher’s note

All claims expressed in this article are solely those of the authors and do not necessarily represent those of their affiliated organizations, or those of the publisher, the editors and the reviewers. Any product that may be evaluated in this article, or claim that may be made by its manufacturer, is not guaranteed or endorsed by the publisher.

## Author disclaimer

This manuscript was prepared using A Case Controlled Etiologic Study of Sarcoidosis (ACCESS) Research Materials obtained from the NHLBI Biologic Specimen and Data Repository Information Coordinating center and does not necessarily reflect the opinions or views of the ACCESS or the NHLBI.

## Supplementary material

Supplementary Table 1 Cohort metadata. **(A)** Table outlining individual characteristics of sarcoidosis cases and controls from ACCESS cohort. **(B)** Table outlining publicly available individual characteristics of Caucasian subjects identified in the GSE19314 cohort (UCSF cohort). **(C)** Table outlining individual characteristics of sarcoidosis cases from the University of Illinois at Chicago Bernie Mac Sarcoidosis Translational Advanced Research Center (UIC STAR) Cohort.

Supplementary Table 2
**(A)** Weighted gene correlation network analysis (WGCNA) module membership. Comprehensive list of all 12,047 genes included in WGCNA analysis and their module membership in relation to disease status (sarcoidosis versus control). Also shown are WGCNA gene significance and module membership parameters for the ACCESS and UCSF cohorts. **(B)** Summary of ACCESS and UCSF cohort module eigengenes per subject.

Supplementary Table 3 Limma-Voom differential expression analysis. Results of moderated t-test between sarcoidosis cases and controls performed on 4,430 genes within WGCNA modules significantly associated with disease in the ACCESS cohort **(A)** and UCSF cohort **(B). (C)** Results of moderated t-test between severe and non-severe sarcoidosis cases in the UIC STAR cohort. Positive log-fold change indicates over-expression in sarcoidosis or severe. Benjamini-Hochberg adjusted (BH.adj) p-values <0.1 were considered significant.

Supplementary Table 4 Module specific significant gene ontology associations. **(A)** Gene ontology and enrichment analysis for magenta module. **(B)** Gene ontology enrichment analysis for blue module. Benjamini-Hochberg adjusted (BH.adj) p-values <0.1 were considered significant. Category is classified by gene ontology relationship with cell proliferation, cell death or other biologic process.

Supplementary Table 5 Magenta (Monocyte Activity Module) and Blue (Lymphocyte Survival) Module Gene Interaction Subnetworks. **(A)** Predicted regulatory interactions between transcription factors and target genes in the Monocyte Activity Module (MAM) “Cell Death” subnetwork shown in **Figure 5A** **(B)** Predicted regulatory interactions between transcription factors and target genes in the Lymphocyte Survival Module (LSM) “Cell Death” subnetwork shown in **Figure 5B**. Gene interaction subnetworks were derived from state specific bipartite networks (sarcoidosis case or control) constructed by the *Monster* algorithm. These subnetworks included transcription factors with significant differential regulatory activity and differentially expressed target genes with greater predicted edge weight in sarcoidosis. Edge weight represents strength of interaction between transcription factor and target gene and is represented as the difference between strength of interaction in case bipartite network and control bipartite network.

Supplementary Table 6 Cell proportions based on statistical deconvolution results. Demonstrates imputed percentages of 15 peripheral blood mononuclear subsets assessed utilizing *Cibersort in-silico* statistical deconvolution of gene expression data from the ACCESS, UCSF, and UIC STAR cohorts with the Immunostates signature gene matrix.

Supplementary Table 7 Partial least squares discriminant analysis. **(A)** Calibration cohort (ACCESS) classification accuracy based on number of correctly and incorrectly classified subjects in each specific transcription factor model on the first principal component. **(B)** Classification accuracy on the UCSF validation cohort determined for each specific transcription factor model based on PLS-DA and receiver operating characteristic (ROC) curve area under the curve (AUC). Comparison to negative control models were performed on ROC curve AUC using Venkatraman’s test with 10,000 bootstraps (p-value <0.05 was considered significant). **(C)** Classification accuracy on the UIC STAR validation cohort determined for TLE3, CBX8, red random, and lightgreen random PLS-DA models based on ROC curve AUC using Venkatraman’s test with 10,000 bootstraps (p-value <0.05 was considered significant). **(D)** Normalized expression of TLE3 and CBX8 target genes as well as red and lightgreen module random gene lists in the UIC STAR cohort. **(E)** Variable importance in projection (VIP) scores for PLS-DA model.

Supplementary Figure 1 Summarized workflow.

Supplementary Figure 2 Normalized gene expression box plots per sample. **(A)** Normalized gene expression in log_2_-counts per million utilizing the trimmed mean of M-values method for normalization and voom with sample quality weights and batch correction for the ACCESS cohort. **(B)** Normalized gene expression using robust multi-array average (log_2_-RMA) for the UCSF cohort **(C)** Normalized gene expression in log_2_-counts per million utilizing the trimmed mean of M-values method for normalization and voom with sample quality weights for the UIC STAR cohort.

Supplementary Figure 3 WGCNA module construction from the ACCESS cohort. **(A)** Dendrogram demonstrating complete-linkage agglomerative hierarchichal clustering of subjects in the ACCESS cohort based on normalized gene expression data. Heatmap below the dendrogram is indicative of individual subject demographic characteristics in this cohort. Gender: female = red, male = white; Age: <30 years old = white, 60-69 years old = red (darker shades red are indicative of older age group); Smoking history: positive smoking history = white, never smoker = red; Disease: control = white, sarcoidosis = red. **(B)** Dendrogram demonstrating complete-linkage hierarchical clustering of genes with dissimilarity based on topological overlap utilizing WGCNA. Height reflects the extent of dissimilarity between genes. Genes are grouped into distinct color-coded modules as shown in the color ribbons beneath the dendrogram. Each module is separated at significant branch points in the dendrogram and consists of a set of interconnected genes identified after adaptive branch pruning (top color ribbon, “Dynamic Tree Cut”). Ultimately, 12 modules were identified after module merging at a maximum dissimilarity of 0.1 given the likelihood of high gene co-expression among closely correlated modules (bottom color ribbon, “Merged Dynamic”).

Supplementary Figure 4 Differential expression analysis in the ACCESS and UCSF (GSE19314) cohorts. **(A, B)** Volcano plots demonstrating differentially expressed genes between sarcoidosis cases and controls within WGCNA gene modules found to be significantly associated with disease (blue, brown, cyan, greenyellow, and magenta) in the ACCESS cohort **(A)** and the UCSF cohort **(B)**. Significance was determined based on false discovery rate <10% (Benjamini-Hochberg adjusted [BH-adj] p-value < 0.1) utilizing the moderated t-test in the limma R-package. Horizontal gold solid line denotes BH-adj p-value of 0.1, vertical gold solid lines denote log_2_-fold change of 1. **(C)** Consensus gene expression between ACCESS and UCSF cohorts was assessed by t-statistics determined for all genes in the blue and magenta modules. **(D, E)** Venn diagrams demonstrate overlap of differentially over-expressed genes in the magenta **(D)** and differentially under-expressed genes in the blue **(E)** modules between cohorts.

Supplementary Figure 5 Differential expression analysis in UIC STAR cohort. **(A)** Volcano plots demonstrating differentially expressed genes between lymphopenic and non-lymphopenic sarcoidosis cases based on clinical CD4+ T-cell grouping. **(B)** Consensus gene expression between ACCESS and UIC STAR cohorts was assessed by t-statistics determined for all genes in the blue and magenta modules.

## References

[1] Ascoli C Huang Y Schott C Turturice BA Metwally A Perkins DL. A circulating micro-RNA signature serves as a diagnostic and prognostic indicator in sarcoidosis. Am J Respir Cell Mol Biol (2017) 58(1):40–54. 10.1165/rcmb.2017-0207OC PMC5941311 28812922

[2] Grunewald J Grutters JC Arkema EV Saketkoo LA Moller DR Müller-Quernheim J. Sarcoidosis. Nat Rev Dis Primers (2019) 5(1):45. doi: 10.1038/s41572-019-0096-x 31273209

[3] Crouser ED Lozanski G Fox CC Hauswirth DW Raveendran R Julian MW. The CD4+ lymphopenic sarcoidosis phenotype is highly responsive to anti-tumor necrosis factor-α therapy. Chest (2010) 137(6):1432–5. doi: 10.1378/chest.09-2576 20525654

[4] Sweiss NJ Salloum R Gandhi S Alegre ML Sawaqed R Badaracco M. Significant CD4, CD8, and CD19 lymphopenia in peripheral blood of sarcoidosis patients correlates with severe disease manifestations. PloS One (2010) 5(2):e9088. 20140091 10.1371/journal.pone.0009088 PMC2816716

[5] Conron M Du Bois RM. Immunological mechanisms in sarcoidosis. Clin Exp Allergy (2001) 31(4):543–54. doi: 10.1046/j.1365-2222.2001.01116.x 11359421

[6] Morell F Levy G Orriols R Ferrer J De Gracia J Sampol G. Delayed cutaneous hypersensitivity tests and lymphopenia as activity markers in sarcoidosis. Chest (2002) 121(4):1239–44. doi: 10.1378/chest.121.4.1239 11948059

[7] Vagts C Ascoli C Fraidenburg DR Baughman RP Huang Y Edafetanure-Ibeh R. Unsupervised clustering reveals sarcoidosis phenotypes marked by a reduction in lymphocytes relate to increased inflammatory activity on 18FDG-PET/CT. Front Med (2021) 8(114). doi: 10.3389/fmed.2021.595077 PMC7943443 33718397

[8] Hawkins C Shaginurova G Shelton DA Herazo-Maya JD Oswald-Richter KA Rotsinger JE. Local and systemic CD4(+) T cell exhaustion reverses with clinical resolution of pulmonary sarcoidosis. J Immunol Res (2017) 2017:3642832. doi: 10.1155/2017/3642832 29234685 PMC5695030

[9] Judson MA. Hepatic, splenic, and gastrointestinal involvement with sarcoidosis. Semin Respir Crit Care Med (2002) 23(6):529–41. doi: 10.1055/s-2002-36517 16088648

[10] Lower EE Smith JT Martelo OJ Baughman RP. The anemia of sarcoidosis. Sarcoidosis (1988) 5(1):51–5. 3381019

[11] Roberts SD Kohli LL Wood KL Wilkes DS Knox KS. CD4+CD28-T cells are expanded in sarcoidosis. Sarcoidosis Vasc Diffuse Lung Dis (2005) 22(1):13–9. 15881275

[12] Hunninghake GW Crystal RG. Pulmonary sarcoidosis: a disorder mediated by excess helper T-lymphocyte activity at sites of disease activity. N Engl J Med (1981) 305(8):429–34. doi: 10.1056/NEJM198108203050804 6454846

[13] Miyara M Amoura Z Parizot C Badoual C Dorgham K Trad S. The immune paradox of sarcoidosis and regulatory T cells. J Exp Med (2006) 203(2):359–70. doi: 10.1084/jem.20050648 PMC2118208 16432251

[14] Broos CE van Nimwegen M Kleinjan A ten Berge B Muskens F in 't Veen JC. Impaired survival of regulatory T cells in pulmonary sarcoidosis. Respir Res (2015) 16:108. doi: 10.1186/s12931-015-0265-8 26376720 PMC4574219

[15] Celada LJ Rotsinger JE Young A Shaginurova G Shelton D Hawkins C. Programmed death-1 inhibition of phosphatidylinositol 3-Kinase/AKT/Mechanistic target of rapamycin signaling impairs sarcoidosis CD4(+) T cell proliferation. Am J Respir Cell Mol Biol (2017) 56(1):74–82. doi: 10.1165/rcmb.2016-0037OC 27564547 PMC5248958

[16] Schott CA Ascoli C Huang Y Perkins DL Finn PW. Declining pulmonary function in interstitial lung disease linked to lymphocyte dysfunction. Am J Respir Crit Care Med (2020) 201(5):610–3. doi: 10.1164/rccm.201910-1909LE PMC7047459 31661301

[17] Mermigkis C Polychonopoulos V Mermigkis D Tsakanika K Heretis M Karagiandis N. Overexpression of BCL-2 protein in bronchoalveolar lavage lymphocytes and macrophages in sarcoidosis. Respiration (2006) 73:221–6. doi: 10.1159/000088688 16195666

[18] Huang H Lu Z Jiang C Liu J Wang Y Xu Z. Imbalance between Th17 and regulatory T-cells in sarcoidosis. Int J Mol Sci (2013) 14(11):21463–73. doi: 10.3390/ijms141121463 PMC3856015 24177566

[19] Agostini C Adami F Semenzato G. New pathogenetic insights into the sarcoid granuloma. Curr Opin Rheumatol (2000) 12(1):71–6. doi: 10.1097/00002281-200001000-00012 10647958

[20] Kunitake R Kuwano K Miyazaki H Nomoto Y Hara N. Apoptosis in the course of granulomatous inflammation in pulmonary sarcoidosis. Eur Respir J (1999) 13:1329–37. doi: 10.1183/09031936.99.13613389 10445608

[21] Chalayer E Bachy E Occelli P Weiler L Faurie P Ghesquieres H. Sarcoidosis and lymphoma: A comparative study. Q J Med (2015) 108(871-878). doi: 10.1093/qjmed/hcv039 25660608

[22] Ozdemir OK Celik G Dalva K Ulger F Elhan A Beksac M. High CD95 expression of BAL lymphocytes predicts chronic course in patients with sarcoidosis. Respirology (2007) 12:869–73. doi: 10.1111/j.1440-1843.2007.01151.x 17986116

[23] Cree IA Nurbhai S Milne G Sawanson Beck J. Cell death in granulomata: the role of apoptosis. J Clin Pathol (1987) 40:1314–9. doi: 10.1136/jcp.40.11.1314 PMC1141231 3320094

[24] Stridh H Planck A Gigliotti D Eklund A Grunewald J. Apoptosis resistant bronchoalveolar lavage (BAL) fluid lymphocytes in sarcoidosis. Thorax (2002) 57:897–901. doi: 10.1136/thorax.57.10.897 12324678 PMC1746191

[25] Rutherford RM Kehren J Staedtler F Chibout SD Egan JJ Tamm M. Functional genomics in sarcoidosis–reduced or increased apoptosis? Swiss Med Wkly (2001) 131(31-32):459–70. 10.4414/smw.2001.09808 11641969

[26] Tana C Giamberardino MA Di Gioacchino M Mezzetti A Schiavone C. Immunopathogenesis of sarcoidosis and risk of malignancy: a lost truth? Int J Immunopathol Pharmacol (2013) 26(2):305–13. doi: 10.1177/039463201302600204 23755746

[27] Oswald-Richter KA Richmond BW Braun NA Isom J Abraham S Taylor TR. Reversal of global CD4+ subset dysfunction is associated with spontaneous clinical resolution of pulmonary sarcoidosis. J Immunol (2013) 190(11):5446–53. doi: 10.4049/jimmunol.1202891 PMC3660530 23630356

[28] Bloom CI Graham CM Berry MP Rozakeas F Redford PS Wang Y. Transcriptional blood signatures distinguish pulmonary tuberculosis, pulmonary sarcoidosis, pneumonias and lung cancers. PloS One (2013) 8(8):e70630. 23940611 10.1371/journal.pone.0070630 PMC3734176

[29] Koth LL Solberg OD Peng JC Bhakta NR Nguyen CP Woodruff PG. Sarcoidosis blood transcriptome reflects lung inflammation and overlaps with tuberculosis. Am J Respir Crit Care Med (2011) 184(10):1153–63. doi: 10.1164/rccm.201106-1143OC PMC3262024 21852540

[30] Maertzdorf J Weiner J Mollenkopf HJ Bauer T Prasse A Müller-Quernheim J. Common patterns and disease-related signatures in tuberculosis and sarcoidosis. Proc Natl Acad Sci U.S.A. (2012) 109(20):7853–8. doi: 10.1073/pnas.1121072109 PMC3356621 22547807

[31] Zhou T Zhang W Sweiss NJ Chen ES Moller DR Knox KS. Peripheral blood gene expression as a novel genomic biomarker in complicated sarcoidosis. PloS One (2012) 7(9):e44818. doi: 10.1371/journal.pone.0044818 22984568 PMC3440319

[32] Monast CS Li K Judson MA Baughman RP Wadman E Watt R. Sarcoidosis extent relates to molecular variability. Clin Exp Immunol (2017) 188(3):444–54. doi: 10.1111/cei.12942 PMC5422855 28205212

[33] Garman L Pelikan RC Rasmussen A Lareau CA Savoy KA Deshmukh US. Single cell transcriptomics implicate novel monocyte and T cell immune dysregulation in sarcoidosis. Front Immunol (2020) 11:567342. doi: 10.3389/fimmu.2020.567342 33363531 PMC7753017

[34] Su R Li MM Bhakta NR Solberg OD Darnell EP Ramstein J. Longitudinal analysis of sarcoidosis blood transcriptomic signatures and disease outcomes. Eur Respir J (2014) 44(4):985–93. doi: 10.1183/09031936.00039714 25142485

[35] Hernández Lemus E Baca López K Lemus R García Herrera R. The role of master regulators in gene regulatory networks. Papers Phys (2015) 7(0):070011. doi: 10.4279/pip.070011

[36] Lambert SA Jolma A Campitelli LF Das PK Yin Y Albu M. The human transcription factors. Cell (2018) 172(4):650–65. doi: 10.1016/j.cell.2018.01.029 PMC12908702 29425488

[37] Lee TI Young RA. Transcriptional regulation and its misregulation in disease. Cell (2013) 152(6):1237–51. doi: 10.1016/j.cell.2013.02.014 PMC3640494 23498934

[38] Pope SD Medzhitov R. Emerging principles of gene expression programs and their regulation. Mol Cell (2018) 71(3):389–97. doi: 10.1016/j.molcel.2018.07.017 30075140

[39] Cheng C Gerstein M. Modeling the relative relationship of transcription factor binding and histone modifications to gene expression levels in mouse embryonic stem cells. Nucleic Acids Res (2012) 40(2):553–68. doi: 10.1093/nar/gkr752 PMC3258143 21926158

[40] Filtz TM Vogel WK Leid M. Regulation of transcription factor activity by interconnected post-translational modifications. Trends Pharmacol Sci (2014) 35(2):76–85. doi: 10.1016/j.tips.2013.11.005 24388790 PMC3954851

[41] Hudson NJ Dalrymple BP Reverter A. Beyond differential expression: the quest for causal mutations and effector molecules. BMC Genomics (2012) 13:356. doi: 10.1186/1471-2164-13-356 22849396 PMC3444927

[42] Tacheny A Dieu M Arnould T Renard P. Mass spectrometry-based identification of proteins interacting with nucleic acids. J Proteomics (2013) 94:89–109. doi: 10.1016/j.jprot.2013.09.011 24060998

[43] Nickles MA Huang K Chang YS Tsoukas MM Sweiss NJ Perkins DL. Gene Co-expression networks identifies common hub genes between cutaneous sarcoidosis and discoid lupus erythematosus. Front Med (Lausanne) (2020) 7:606461. doi: 10.3389/fmed.2020.606461 33324666 PMC7724034

[44] ACCESS Research Group. Design of a case control etiologic study of sarcoidosis (ACCESS). J Clin Epidemiol (1999) 52(12):1173–86. 10.1016/s0895-4356(99)00142-0 10580780

[45] Barrett T Wilhite SE Ledoux P Evangelista C Kim IF Tomashevsky M. NCBI GEO: archive for functional genomics data sets–update. Nucleic Acids Res (2013) 41(Database issue):D991–5. 10.1093/nar/gks1193 PMC3531084 23193258

[46] Langfelder P Horvath S. WGCNA: an r package for weighted correlation network analysis. BMC Bioinf (2008) 9:559. doi: 10.1186/1471-2105-9-559 PMC2631488 19114008

[47] Team RC. R: A language and environment for statistical computing. Vienna, Austria: R Foundation for Statistical Computing (2016).

[48] Langfelder P Luo R Oldham MC Horvath S. Is my network module preserved and reproducible? PloS Comput Biol (2011) 7(1):e1001057. doi: 10.1371/journal.pcbi.1001057 21283776 PMC3024255

[49] Ritchie ME Phipson B Wu D Hu Y Law CW Shi W. Limma powers differential expression analyses for RNA-sequencing and microarray studies. Nucleic Acids Res (2015) 43(7):e47. doi: 10.1093/nar/gkv007 25605792 PMC4402510

[50] Huang da W Sherman BT Lempicki RA. Systematic and integrative analysis of large gene lists using DAVID bioinformatics resources. Nat Protoc (2009) 4(1):44–57. 19131956 10.1038/nprot.2008.211

[51] Huang da W Sherman BT Lempicki RA. Bioinformatics enrichment tools: paths toward the comprehensive functional analysis of large gene lists. Nucleic Acids Res (2009) 37(1):1–13. 19033363 10.1093/nar/gkn923 PMC2615629

[52] Monaco G Lee B Xu W Mustafah S Hwang YY Carré C. RNA-Seq signatures normalized by mRNA abundance allow absolute deconvolution of human immune cell types. Cell Rep (2019) 26(6):1627–1640.e7. doi: 10.1016/j.celrep.2019.01.041 30726743 PMC6367568

[53] Uhlen M Karlsson MJ Zhong W Tebani A Pou C Mikes J. A genome-wide transcriptomic analysis of protein-coding genes in human blood cells. Science (2019) 366(6472). doi: 10.1126/science.aax9198 31857451

[54] Schlauch D Glass K Hersh CP Silverman EK Quackenbush J. Estimating drivers of cell state transitions using gene regulatory network models. BMC Syst Biol (2017) 11(1):139. doi: 10.1186/s12918-017-0517-y 29237467 PMC5729420

[55] Jalili M Salehzadeh-Yazdi A Asgari Y Arab SS Yaghmaie M Ghavamzadeh A. CentiServer: A comprehensive resource, web-based application and r package for centrality analysis. PloS One (2015) 10(11):e0143111. doi: 10.1371/journal.pone.0143111 26571275 PMC4646361

[56] Qi X Fuller E Wu Q Wu Y Zhang C-Q. Laplacian centrality: A new centrality measure for weighted networks. Inf Sci (2012) 194:240–53. doi: 10.1016/j.ins.2011.12.027

[57] Newman AM Liu CL Green MR Gentles AJ Feng W Xu Y. Robust enumeration of cell subsets from tissue expression profiles. Nat Methods (2015) 12(5):453–7. doi: 10.1038/nmeth.3337 PMC4739640 25822800

[58] Kucheryavskiy S. Mdatools - r package for chemometrics. Chemometrics Intelligent Lab Syst (2020) 198. doi: 10.1016/j.chemolab.2020.103937

[59] Robin X Turck N Hainard A Tiberti N Lisacek F Sanchez JC. pROC: an open-source package for r and s+ to analyze and compare ROC curves. BMC Bioinf (2011) 12:77. doi: 10.1186/1471-2105-12-77 PMC3068975 21414208

[60] Chong I-G Jun C-H. Performance of some variable selection methods when multicollinearity is present. Chemometrics Intelligent Lab Syst (2005) 78:103–12. doi: 10.1016/j.chemolab.2004.12.011

[61] Kendrick YR. Transcriptomic approach to understanding and characterising disease pathogenesis in sarcoidosis. England, United Kingdom: University of Oxford (2017).

[62] Rosenbaum JT Choi D Wilson DJ Grossniklaus HE Harrington CA Sibley CH. Parallel gene expression changes in sarcoidosis involving the lacrimal gland, orbital tissue, or blood. JAMA Ophthalmol (2015) 133(7):770–7. doi: 10.1001/jamaophthalmol.2015.0726 PMC5021543 25880323

[63] Blankley S Graham CM Turner J Berry MP Bloom CI Xu Z. The transcriptional signature of active tuberculosis reflects symptom status in extra-pulmonary and pulmonary tuberculosis. PloS One (2016) 11(10):e0162220. doi: 10.1371/journal.pone.0162220 27706152 PMC5051928

[64] Scott MKD Quinn K Li Q Carroll R Warsinske H Vallania F. Increased monocyte count as a cellular biomarker for poor outcomes in fibrotic diseases: a retrospective, multicentre cohort study. Lancet Respir Med (2019) 7(6):497–508. doi: 10.1016/S2213-2600(18)30508-3 30935881 PMC6529612

[65] Yi JS Rosa-Bray M Staats J Zakroysky P Chan C Russo MA. Establishment of normative ranges of the healthy human immune system with comprehensive polychromatic flow cytometry profiling. PloS One (2019) 14(12):e0225512. doi: 10.1371/journal.pone.0225512 31825961 PMC6905525

[66] Bhargava M Viken KJ Barkes B Griffin TJ Gillespie M Jagtap PD. Novel protein pathways in development and progression of pulmonary sarcoidosis. Sci Rep (2020) 10(1):13282. doi: 10.1038/s41598-020-69281-8 32764642 PMC7413390

[67] Drent M. Association of heterozygote glucose-6-phosphate-dehydrogenase deficiency with more advanced disease in sarcoidosis. Sarcoidosis Vasc Diffuse Lung Dis (1999) 16(1):108–9. 10207951

[68] Celada LJ Kropski JA Herazo-Maya JD Luo W Creecy A Abad AT. PD-1 up-regulation on CD4(+) T cells promotes pulmonary fibrosis through STAT3-mediated IL-17A and TGF-β1 production. Sci Transl Med (2018) 10(460). doi: 10.1126/scitranslmed.aar8356 PMC6263177 30257954

[69] Braun NA Celada LJ Herazo-Maya JD Abraham S Shaginurova G Sevin CM. Blockade of the programmed death-1 pathway restores sarcoidosis CD4(+) T-cell proliferative capacity. Am J Respir Crit Care Med (2014) 190(5):560–71. doi: 10.1164/rccm.201401-0188OC PMC4214083 25073001

[70] Bhatla D Gerbing RB Alonzo TA Mehta PA Deal K Elliott J. DNA Repair polymorphisms and outcome of chemotherapy for acute myelogenous leukemia: a report from the children’s oncology group. Leukemia (2008) 22(2):265–72. doi: 10.1038/sj.leu.2405000 PMC2914507 18033323

[71] Esteves T Aparicio G Garcia-Patos V. Is there any association between sarcoidosis and infectious agents?: a systematic review and meta-analysis. BMC Pulm Med (2016) 16(1):165. doi: 10.1186/s12890-016-0332-z 27894280 PMC5126827

[72] Goldberg HJ Fiedler D Webb A Jagirdar J Hoyumpa AM Peters J. Sarcoidosis after treatment with interferon-alpha: a case series and review of the literature. Respir Med (2006) 100(11):2063–8. doi: 10.1016/j.rmed.2006.03.004 16675213

[73] Mostafavi S Yoshida H Moodley D LeBoité H Rothamel K Raj T. Parsing the interferon transcriptional network and its disease associations. Cell (2016) 164(3):564–78. doi: 10.1016/j.cell.2015.12.032 PMC4743492 26824662

[74] Neveu B Spinella JF Richer C Lagacé K Cassart P Lajoie M. CLIC5: a novel ETV6 target gene in childhood acute lymphoblastic leukemia. Haematologica (2016) 101(12):1534–43. doi: 10.3324/haematol.2016.149740 PMC5479611 27540136

[75] Pierce AJ Johnson RD Thompson LH Jasin M. XRCC3 promotes homology-directed repair of DNA damage in mammalian cells. Genes Dev (1999) 13(20):2633–8. doi: 10.1101/gad.13.20.2633 PMC317094 10541549

[76] Popanda O Schattenberg T Phong CT Butkiewicz D Risch A Edler L. Specific combinations of DNA repair gene variants and increased risk for non-small cell lung cancer. Carcinogenesis (2004) 25(12):2433–41. doi: 10.1093/carcin/bgh264 15333465

[77] Rothenberg EV Hosokawa H Ungerbäck J. Mechanisms of action of hematopoietic transcription factor PU.1 in initiation of T-cell development. Front Immunol (2019) 10:228. doi: 10.3389/fimmu.2019.00228 30842770 PMC6391351

[78] Sweiss NJ Zhang W Franek BS Kariuki SN Moller DR Patterson KC. Linkage of type I interferon activity and TNF-alpha levels in serum with sarcoidosis manifestations and ancestry. PloS One (2011) 6(12):e29126. doi: 10.1371/journal.pone.0029126 22195005 PMC3237595

[79] Zakrzewska A Cui C Stockhammer OW Benard EL Spaink HP Meijer AH. Macrophage-specific gene functions in Spi1-directed innate immunity. Blood (2010) 116(3):e1–11. doi: 10.1182/blood-2010-01-262873 20424185

[80] Ziegenhagen MW Müller-Quernheim J. The cytokine network in sarcoidosis and its clinical relevance. J Intern Med (2003) 253(1):18–30. doi: 10.1046/j.1365-2796.2003.01074.x 12588535

[81] Klauke K Radulović V Broekhuis M Weersing E Zwart E Olthof S. Polycomb cbx family members mediate the balance between haematopoietic stem cell self-renewal and differentiation. Nat Cell Biol (2013) 15(4):353–62. doi: 10.1038/ncb2701 23502315

[82] Tan J Jones M Koseki H Nakayama M Muntean AG Maillard I. CBX8, a polycomb group protein, is essential for MLL-AF9-induced leukemogenesis. Cancer Cell (2011) 20(5):563–75. doi: 10.1016/j.ccr.2011.09.008 PMC3220883 22094252

[83] Radulović V de Haan G Klauke K. Polycomb-group proteins in hematopoietic stem cell regulation and hematopoietic neoplasms. Leukemia (2013) 27(3):523–33. doi: 10.1038/leu.2012.368 23257781

[84] Abo Al Hayja M Eklund A Grunewald J Wahlström J. Reduced expression of peroxisome proliferator-activated receptor alpha in BAL and blood T cells of non-löfgren’s sarcoidosis patients. J Inflamm (Lond) (2015) 12:28. 25969669 10.1186/s12950-015-0071-6 PMC4428503

[85] Villanueva CJ Waki H Godio C Nielsen R Chou WL Vargas L. TLE3 is a dual-function transcriptional coregulator of adipogenesis. Cell Metab (2011) 13(4):413–27. doi: 10.1016/j.cmet.2011.02.014 PMC3089971 21459326

[86] Culver DA Barna BP Raychaudhuri B Bonfield TL Abraham S Malur A. Peroxisome proliferator-activated receptor gamma activity is deficient in alveolar macrophages in pulmonary sarcoidosis. Am J Respir Cell Mol Biol (2004) 30(1):1–5. 14512375 10.1165/rcmb.2003-0304RC

[87] Patel S Alam A Pant R Chattopadhyay S. Wnt signaling and its significance within the tumor microenvironment: Novel therapeutic insights. Front Immunol (2019) 10:2872. doi: 10.3389/fimmu.2019.02872 31921137 PMC6927425

[88] Ramakrishnan AB Sinha A Fan VB Cadigan KM. The wnt transcriptional switch: TLE removal or inactivation? Bioessays (2018) 40(2). doi: 10.1002/bies.201700162 PMC7495026 29250807

[89] Ljungberg JK Kling JC Tran TT Blumenthal A. Functions of the WNT signaling network in shaping host responses to infection. Front Immunol (2019) 10:2521. doi: 10.3389/fimmu.2019.02521 31781093 PMC6857519

[90] Levänen B Wheelock AM Eklund A Grunewald J Nord M. Increased pulmonary wnt (wingless/integrated)-signaling in patients with sarcoidosis. Respir Med (2011) 105(2):282–91. doi: 10.1016/j.rmed.2010.11.018 21146388

[91] Crouser ED Julian MW Crawford M Shao G Yu L Planck SR. Differential expression of microRNA and predicted targets in pulmonary sarcoidosis. Biochem Biophys Res Commun (2012) 417(2):886–91. doi: 10.1016/j.bbrc.2011.12.068 PMC4041713 22209793

[92] Saeidi A Zandi K Cheok YY Saeidi H Wong WF Lee CYQ. T-Cell exhaustion in chronic infections: Reversing the state of exhaustion and reinvigorating optimal protective immune responses. Front Immunol (2018) 9:2569. doi: 10.3389/fimmu.2018.02569 30473697 PMC6237934

[93] Tang L Zhang Y Hu Y Mei H. T Cell exhaustion and CAR-T immunotherapy in hematological malignancies. BioMed Res Int (2021) 2021:6616391. doi: 10.1155/2021/6616391 33728333 PMC7936901

[94] Chen HY Wu YF Chou FC Wu YH Yeh LT Lin KI. Intracellular galectin-9 enhances proximal TCR signaling and potentiates autoimmune diseases. J Immunol (2020) 204(5):1158–72. doi: 10.4049/jimmunol.1901114 31969388

[95] Dai SY Nakagawa R Itoh A Murakami H Kashio Y Abe H. Galectin-9 induces maturation of human monocyte-derived dendritic cells. J Immunol (2005) 175(5):2974–81. doi: 10.4049/jimmunol.175.5.2974 16116184

[96] Gooden MJ Wiersma VR Samplonius DF Gerssen J van Ginkel RJ Nijman HW. Galectin-9 activates and expands human T-helper 1 cells. PloS One (2013) 8(5):e65616. doi: 10.1371/journal.pone.0065616 23741502 PMC3669208

[97] Su EW Bi S Kane LP. Galectin-9 regulates T helper cell function independently of Tim-3. Glycobiology (2011) 21(10):1258–65. doi: 10.1093/glycob/cwq214 PMC3167474 21187321

[98] Yang RY Hsu DK Liu FT. Expression of galectin-3 modulates T-cell growth and apoptosis. Proc Natl Acad Sci U.S.A. (1996) 93(13):6737–42. doi: 10.1073/pnas.93.13.6737 PMC39096 8692888

[99] Kouo T Huang L Pucsek AB Cao M Solt S Armstrong T. Galectin-3 shapes antitumor immune responses by suppressing CD8+ T cells via LAG-3 and inhibiting expansion of plasmacytoid dendritic cells. Cancer Immunol Res (2015) 3(4):412–23. doi: 10.1158/2326-6066.CIR-14-0150 PMC4390508 25691328

[100] Vogt S Trendelenburg M Tamm M Stolz D Hostettler KE Osthoff M. Local and systemic concentrations of pattern recognition receptors of the lectin pathway of complement in a cohort of patients with interstitial lung diseases. Front Immunol (2020) 11(2412). doi: 10.3389/fimmu.2020.562564 PMC7546814 33101280

[101] Ho LP Urban BC Thickett DR Davies RJ McMichael AJ. Deficiency of a subset of T-cells with immunoregulatory properties in sarcoidosis. Lancet (2005) 365(9464):1062–72. doi: 10.1016/S0140-6736(05)71143-0 15781102

[102] Yordy JS Muise-Helmericks RC. Signal transduction and the ets family of transcription factors. Oncogene (2000) 19(55):6503–13. doi: 10.1038/sj.onc.1204036 11175366

[103] Dubaniewicz A Typiak M Wybieralska M Szadurska M Nowakowski S Staniewicz-Panasik A. Changed phagocytic activity and pattern of fcγ and complement receptors on blood monocytes in sarcoidosis. Hum Immunol (2012) 73(8):788–94. doi: 10.1016/j.humimm.2012.05.005 22609476

[104] Rossman MD Chien P Cassizzi A Elias JA Schreiber AD. Increased monocyte Fc(IgG) receptor expression in sarcoidosis. Ann N Y Acad Sci (1986) 465:260–7. doi: 10.1111/j.1749-6632.1986.tb18502.x 3460381

[105] Schnerch J Prasse A Vlachakis D Schuchardt KL Pechkovsky DV Goldmann T. Functional toll-like receptor 9 expression and CXCR3 ligand release in pulmonary sarcoidosis. Am J Respir Cell Mol Biol (2016) 55(5):749–57. doi: 10.1165/rcmb.2015-0278OC 27390897

[106] Pacheco Y Lim CX Weichhart T Valeyre D Bentaher A Calender A. Sarcoidosis and the mTOR, Rac1, and autophagy triad. Trends Immunol (2020) 41(4):286–99. doi: 10.1016/j.it.2020.01.007 32122794

[107] Sanna F Bonatesta RR Frongia B Uda S Banni S Melis MP. Production of inflammatory molecules in peripheral blood mononuclear cells from severely glucose-6-phosphate dehydrogenase-deficient subjects. J Vasc Res (2007) 44(4):253–63. doi: 10.1159/000100903 17361089

[108] Zenarruzabeitia O Vitallé J Eguizabal C Simhadri VR Borrego F. The biology and disease relevance of CD300a, an inhibitory receptor for phosphatidylserine and phosphatidylethanolamine. J Immunol (2015) 194(11):5053–60. doi: 10.4049/jimmunol.1500304 25980030

[109] Metcalf TU Wilkinson PA Cameron MJ Ghneim K Chiang C Wertheimer AM. Human monocyte subsets are transcriptionally and functionally altered in aging in response to pattern recognition receptor agonists. J Immunol (2017) 199(4):1405–17. doi: 10.4049/jimmunol.1700148 PMC5548610 28696254

[110] Sanlioglu S Williams CM Samavati L Butler NS Wang G McCray PB Jr.. Lipopolysaccharide induces Rac1-dependent reactive oxygen species formation and coordinates tumor necrosis factor-alpha secretion through IKK regulation of NF-kappa b. J Biol Chem (2001) 276(32):30188–98. doi: 10.1074/jbc.M102061200 11402028

[111] Agostini C Trentin L Facco M Sancetta R Cerutti A Tassinari C. Role of IL-15, IL-2, and their receptors in the development of T cell alveolitis in pulmonary sarcoidosis. J Immunol (1996) 157(2):910–8. 8752945

[112] Allard-Chamard H Mishra HK Nandi M Mayhue M Menendez A Ilangumaran S. Interleukin-15 in autoimmunity. Cytokine (2020) 136:155258. doi: 10.1016/j.cyto.2020.155258 32919253

[113] Brandsma AM Schwartz SL Wester MJ Valley CC Blezer GLA Vidarsson G. Mechanisms of inside-out signaling of the high-affinity IgG receptor FcγRI. Sci Signal (2018) 11(540). doi: 10.1126/scisignal.aaq0891 PMC7521114 30042128

[114] Kanazawa N Okafuji I Kambe N Nishikomori R Nakata-Hizume M Nagai S. Early-onset sarcoidosis and CARD15 mutations with constitutive nuclear factor-kappaB activation: common genetic etiology with blau syndrome. Blood (2005) 105(3):1195–7. 10.1182/blood-2004-07-2972 15459013

[115] Kim DS Paik SH Lim CM Lee SD Koh Y Kim WS. Value of ICAM-1 expression and soluble ICAM-1 level as a marker of activity in sarcoidosis. Chest (1999) 115(4):1059–65. doi: 10.1378/chest.115.4.1059 10208208

[116] Negroni A Pierdomenico M Cucchiara S Stronati L. NOD2 and inflammation: current insights. J Inflamm Res (2018) 11:49–60. doi: 10.2147/JIR.S137606 29483781 PMC5813767

[117] Sato H Williams HR Spagnolo P Abdallah A Ahmad T Orchard TR. CARD15/NOD2 polymorphisms are associated with severe pulmonary sarcoidosis. Eur Respir J (2010) 35(2):324–30. doi: 10.1183/09031936.00010209 19679608

[118] Webster JD Vucic D. The balance of TNF mediated pathways regulates inflammatory cell death signaling in healthy and diseased tissues. Front Cell Dev Biol (2020) 8:365. doi: 10.3389/fcell.2020.00365 32671059 PMC7326080

[119] Lepzien R Liu S Czarnewski P Nie M Österberg B Baharom F. Monocytes in sarcoidosis are potent TNF producers and predict disease outcome. Eur Respir J (2021). doi: 10.1183/13993003.03468-2020 PMC8295505 33446605

[120] Sutherland JS Loxton AG Haks MC Kassa D Ambrose L Lee JS. Differential gene expression of activating fcγ receptor classifies active tuberculosis regardless of human immunodeficiency virus status or ethnicity. Clin Microbiol Infect (2014) 20(4):O230–8. doi: 10.1111/1469-0691.12383 24205913

[121] Vogelpoel LT Baeten DL de Jong EC den Dunnen J. Control of cytokine production by human fc gamma receptors: implications for pathogen defense and autoimmunity. Front Immunol (2015) 6:79. doi: 10.3389/fimmu.2015.00079 25759693 PMC4338787

[122] Xu JL Guo Y. FCGR1A serves as a novel biomarker and correlates with immune infiltration in four cancer types. Front Mol Biosci (2020) 7:581615. doi: 10.3389/fmolb.2020.581615 33344503 PMC7744780

[123] van der Poel CE Spaapen RM van de Winkel JG Leusen JH. Functional characteristics of the high affinity IgG receptor, FcγRI. J Immunol (2011) 186(5):2699–704. doi: 10.4049/jimmunol.1003526 21325219

[124] Linke M Pham HT Katholnig K Schnöller T Miller A Demel F. Chronic signaling via the metabolic checkpoint kinase mTORC1 induces macrophage granuloma formation and marks sarcoidosis progression. Nat Immunol (2017) 18(3):293–302. doi: 10.1038/ni.3655 28092373 PMC5321578

