# Supplementary material for: Altered transcription factor targeting is associated with differential peripheral blood mononuclear cell proportions in sarcoidosis
Source: Front Immunol. 2022 Oct 13;13:848759. doi: 10.3389/fimmu.2022.848759 (PMC9608777; doi:10.3389/fimmu.2022.848759)
Supplement: Supplementary file 2 [file DataSheet_2.docx]

**Title:** Altered Transcription Factor Targeting is Associated with Differential Peripheral Blood Mononuclear Cell Proportions in Sarcoidosis

**Authors:** Christian Ascoli, MD^1†^; Cody A. Schott, MD PhD^2†^; Yue Huang, PhD^1†^; Benjamin A. Turturice, MD PhD^2^; Wangfei Wang, MS^3^; Naomi Ecanow, BS^2^; Nadera J. Sweiss, MD^1,4^; David L. Perkins, MD PhD^5‡^; Patricia W. Finn, MD^1‡^; on behalf of the ACCESS Research Group*

**Institutions:**

1. Division of Pulmonary, Critical Care, Sleep, and Allergy, Department of Medicine, University of Illinois at Chicago, Chicago, IL, United States 2. University of Illinois at Chicago College of Medicine, Chicago, IL, United States 3. University of Illinois at Chicago College of Engineering and Medicine, Department of Bioengineering, Chicago, IL, United States 4. Division of Rheumatology, Department of Medicine, University of Illinois at Chicago, Chicago, IL, United States 5. Division of Nephrology, Department of Medicine, University of Illinois at Chicago, Chicago, IL, United States

† These authors contributed equally and should be considered co-first authors.

^‡^ These authors contributed equally to this work and should be considered co-last authors.

* *The ACCESS Research Group as outlined in Appendix E*

**Data Supplement**

*Cilnical Characteristics and Messenger RNA-seq (mRNA-seq) Library Preparation and Annotation for the ACCESS Cohort*

The study design and subject enrollment for ACCESS (A Case Controlled Etiologic Study of Sarcoidosis; ClinicaTrials.gov identifier: NCT00005276) were previously described [1]. Baseline characteristics of the ACCESS cohort were obtained from results reported on ACCESS study data forms based on standardization data acquisition described in the ACCESS study procedures manual. Baseline characteristics of the ACCESS cohort are summarized in **Supplemental Table S1A**. Definitive organ involvement refers to proof of features typical for sarcoidosis granulomas on biopsy (upon ruling out alternate causes of granulomas) or additional criteria based on the ACCESS study procedures manual, volume I Table 3-1A (<https://biolincc.nhlbi.nih.gov/studies/access/>). In the case of pulmonary sarcoidosis, definitive involvement could also be determined by restrictive pulmonary function tests or chest radiograph with bilateral hilar adenopathy, diffuse infiltrates, or upper lobe fibrosis.

Gene expression of peripheral blood mononuclear cells (PBMCs) involved in sarcoidosis utilizing whole transcriptome shotgun sequencing of messenger RNA (mRNA-seq) was investigated. PBMCs were isolated from whole blood collected in heparinized collection tubes via a standard Ficoll-hypaque density gradient from all sarcoidosis cases and controls (n=28) [2]. Upon obtaining PBMCs, RNA was isolated utilizing the guanidinium thiocyanate phenol-chloroform extraction method and stored at -80^o^C until mRNA-seq preparation [3]. The quality and quantity of total RNA isolated from PBMCs from all samples was analyzed with the RNA 6000 Pico Kit on the 2100 Bioanalyzer Instrument *(Agilent Technologies, Catalog #: 5067-1513)* [4, 5]. Samples with an RNA Integrity Number (RIN) of 6 or greater were included in the study. Transcriptomic libraries were prepared with the Illumina TruSeq Stranded mRNA Sample Preparation kit as directed by the manufacturer for the low sample protocol (*Illumina Inc. Catalog #: 20020594*). Each library was quantified using the Qubit 2.0 fluorometer (*ThermoFisher Scientific Inc.*). Assessment of individual library quality was based on concentration and conducted on the 2100 Bioanalyzer Instrument with either the High Sensitivity or 7500 DNA Assay kits (*Agilent Technologies, Catalog #: 5067-4626 and 5067-1506*). Library size selection was performed using 2% Agarose Gel Cassette (*Pippin Prep, Sage Science Inc.*) when necessary. Individually barcoded mRNA-seq libraries were pooled into batches of 8-12 samples and sequenced on the Illumina MiSeq platform (Illumina, Inc.) at 2 x 151 paired-end reads (*Illumina Inc. MiSeq Reagent Kit V3-600, Catalog #: MS-102-3003*) at a final pooled library concentration of 8pM. Upon completion of sequencing, base calls were converted into sequence data and paired-end FASTQ files were generated. The quality of sequencing reads was then assessed from FASTQ files using FastQC and followed by removal of adapter sequences by *Cutadapt* and reassessment of quality with FastQC [6, 7]. All samples demonstrated a PHRED quality score greater than 30 and were considered suitable for downstream analysis. FASTQ-formatted reads were aligned and quantified using *Salmon* against the human reference genome (Ensembl GRCh38.p12 release 94) indexed at the transcript level and aggregated to then be annotated at specific gene levels utilizing *Tximport* (R bioconductor package) for a total of 37,788 annotated genes [8-10]. Gene counts estimated from the *Salmon* quasi-alignments by *Tximport* were utilized for downstream analyses unless otherwise specified. All raw sequencing data for the ACCESS cohort has been deposited to GEO, series record GSE155644.

*Processing of Microarray Data for the UCSF Cohort*

Publicly available gene expression data (CEL files) obtained with *Affymetrix Human Genome U133 Plus 2.0 Array* [HG-U133_Plus_2] chips from peripheral blood mononuclear cells and associated metadata was downloaded from the gene expression omnibus (GEO) database series GSE19314 (UCSF Sarcoidosis and Hypersensitivity Pneumonitis Cohort) [11, 12]. While the initial study included patients with hypersensitivity pneumonitis as well as subjects from various ethnic/racial backgrounds, only Caucasians with sarcoidosis were considered for this study to specifically assess sarcoidosis and reduce genotypic heterogeneity for statistical validation. Metadata for selected Caucasian sarcoidosis cases (n=31) and controls (n=16) as reported for GSE19314 is summarized in **Supplemental** **Table S1B**. Evaluation of gene expression data was performed by reading CEL files with measured gene probe intensities and locations for hybridized arrays into the R Statistical Environment (version 3.5) with the *affy* R bioconductor package and transformed utilizing the robust multiarray average normalization (RMA) method to obtain relative log_2_ expression values (**log_2_-RMA)** [13, 14]. Affymetrix probe identifiers were then paired to corresponding unique Ensembl gene identifiers and annotated. Redundancy was reduced by applying the “MaxMean” method in the *collapserows* function from the *WGCNA* R package resulting in 21,869 unique genes [15-17]. Unique genes from the UCSF cohort microarray data where then matched to corresponding genes identified in the ACCESS cohort resulting in 18,653 genes. The microarray gene set was reduced based on the ACCESS mRNA-seq filtering for low median counts resulting in 12,047 genes with only 602 (5%) of genes demonstrating a low median intensity in the range of background intensity (≤ 4). Batch effects for microarray data in the UCSF cohort were accounted for with the *removeBatchEffect* function in the *limma* bioconductor package prior to *WGCNA* analysis and differential expression of genes within significant and preserved modules between the ACCESS and UCSF cohorts was assessed between sarcoidosis cases and controls utilizing a moderated t-statistic. Findings were deemed significant at a predetermined false discovery rate (FDR) of 10% (*Benjamini-Hochberg adjusted* [*BH.adj*] *p-value* <0.1) (**Supplemental** **Table S3B, Supplemental Figure S4B**) [18].

*Weighted Gene Co-expression Analysis*

Weighted gene co-expression network analysis (*WGCNA*) in the R Statistical Environment was utilized to identify gene modules (clusters) and their relationship with clinical features [13, 17]. Given differences in RNA sequencing platforms, prior to *WGCNA* analysis, unique genes identified by mRNA-seq from the ACCESS cohort were matched to corresponding Ensembl gene identifiers within the UCSF microarray gene probe set to establish a uniform gene expression dataset. Matching yielded a total of 18,653 genes common to both cohorts. Sparse gene expression data was then filtered based on mRNA-seq counts from the ACCESS cohort utilizing an arbitrary cutoff to exclude those genes with a median count ≤ 1 across all samples. Filtered mRNA-seq count data resulted in 12,047 genes and was normalized via the trimmed mean of M-values (TMM) method and transformed to log_2_-counts per million by using *voom with sample quality weights* to down-weight outlier samples followed by removal of batch effects as described in the *limma* bioconductor package (functions: *voomWithQualityWeights* and *removeBatchEffect*) to perform *WGCNA* analysis (**Supplemental Figure S2A**) [18, 19]. For the UCSF cohort, relative robust multi-array average expression (**log_2_-RMA) normalization** of filtered genes matched to the ACCESS cohort was utilized for *WGCNA* analysis after processing of raw microarray expression data (**Supplemental Figure S2B**).

The *WGCNA* stepwise approach was then utilized to construct the signed gene co-expression network for the ACCESS cohort. No subjects were considered outliers by complete-linkage agglomerative hierarchical clustering and network construction was carried out on gene expression data from the 12,047 filtered genes from all subjects (**Supplemental Figure S3A**). To specify the network, adjacency was determined by utilizing the biweight midcorrelation to account for outliers and establish gene co-expression similarity along with a soft-thresholding power of 9 to assign connection weights. Gene modules were then established by coupling topological overlap measure (TOM) dissimilarity with mean connectivity denominator and complete linkage hierarchical clustering after adaptive branch pruning (*cutreeDynamic* with minimum module size of 240 and *deepSplit =3*). Modules with similar expression profiles after adaptive branch pruning were then merged given the likelihood of high gene co-expression among them at a maximum dissimilarity of 0.1 (corresponding to a correlation of 0.9) (**Supplemental Figure S3B**). Module-trait relationships determined by *WGCNA* are demonstrated in **Figure 1A** and a comprehensive list of module specific genes is found in **Supplemental Table S2A**.

To determine resemblance of gene expression modules between sarcoidosis cohorts, the *modulePreservation* function in the *WGCNA* package was utilized to calculate the Z-summary composite preservation statistic for signed networks with the biweight midcorrelation and 10,000 permutations after pre-specifying gene expression data from the ACCESS cohort as the reference data set. Gene modules identified in the ACCESS co-expression network with Z-summary scores, representative of the average of *Z*-scores computed for density and connectivity measures, greater than 10 were deemed to have strong evidence of preservation between cohorts and considered for further analysis (**Figure 1B**) [17, 20].

Module eigengenes (**Supplemental Table S2B**), derived from standardized gene expression within the module, were extracted as representations of modules within a sample and associated with clinical features. In addition to associations between gene modules and clinical features, the association between gene modules and mRNA-seq batch was also explored. None of the twelve modules was found to be significantly associated with batch (Spearman’s correlation *p-value* >0.05).

*WGCNA Module PBMC Subset Enrichment*

To determined *WGCNA* module PBMC subset enrichment, cellular gene expression data from 109 blood cell samples was obtained from the **file “**rna_blood_cell_sample_tpm_m.tsv.zip” downloaded from the Human Protein Atlas (version 20.1, <https://www.proteinatlas.org>). Protein coding Transcripts per Million (pTPM) based on Ensembl version 92.38 were normalized by the addition of a 0.05 pseudocount (1/2 minimum) and log_2_ transformed. Gene subsets were determined by *WGCNA* module assignment. Normalized pTPM were modeled as a function of the fixed effect cell type and the random effect gene in a mixed effect module. Reference was set to total PBMC [21, 22]. Kruskal-Wallis (KW) one-way analysis of variance test followed by *post hoc* analysis using Dunn’s test with Benjamini-Hochberg adjustment to account for multiplicity were utilized to assess differences in PBMC subset enrichment (p-values < 0.05 and < 0.1 were pre-specified as significant for KW and Dunn’s tests; respectively).

*Monster Algorithm*

To identify master regulator transcription factors capable of driving the transition from the healthy to the dysregulated immune response observed in sarcoidosis we applied the *Monster* (Modeling network state transitions from expression and regulatory data) algorithm in the R Statistical Environment to significant and preserved gene co-expression modules [23]. *Monster* is a regression-based method for inferring master regulators that drive cell states at the gene regulatory network level. It builds on state specific unidirectional interactions between transcription factors and their target genes based on direct and indirect evidence of interplay to estimate changes in transcription factor targeting patterns. As a result, it discriminates between transcription factors that maintain unchanged targeting patterns and cell state transition regulators that experience change in targeting patterns within a gene regulatory network and defines differential transcription factor involvement (DTFI) as the magnitude of targeting change.

To determine significant DTFI, ACCESS cohort control samples were defined as the baseline (reference) state and sarcoidosis samples as the perturbed (final) state. Subsequently, the change in strength of interaction between transcription factors and target genes was predicted from unidirectional (bipartite) networks and a “regulatory network prior.” For this purpose, a transformation matrix was generated utilizing the *monster* function with 10,000 randomized permutations in addition to the default bipartite edge reconstruction from expression data algorithm (“bere”) and default direct versus indirect evidence weight parameter (alpha=0.5). Gene expression data for bipartite networks was derived from the ACCESS cohort *WGCNA* gene modules significantly associated with disease and preserved in the UCSF cohort.

The regulatory network prior in the *Monster* algorithm is a baseline set of regulatory interactions for each predefined group (i.e., sarcoidosis cases and matched controls) derived from established transcription factor interactions with DNA sequence binding motifs. To construct this network, we utilized the Gene Transcription Regulation Database (GTRD v.19.10; <http://gtrd.biouml.org/>) to obtain data relating to known protein interactions with DNA determined by chromatin immunoprecipitation sequencing (ChIP-seq) [24]. First, Uniprot accession identifiers for all 1,333 human proteins with target genes listed in GTRD were converted to corresponding Ensembl reference gene identifiers on the Database for Annotation, Visualization and Integrated Discovery (DAVID Bioinformatics Database v6.8; <https://david.ncifcrf.gov>) [25-27]. Subsequently, to determine transcription factors of interest, genes within the ACCESS cohort *WGCNA* modules significantly associated with disease and preserved in the UCSF cohort were matched against transcription factors identified in GTRD. Conversely, genes within these modules were considered transcription factor targets if at least one protein-DNA interaction peak was identified under various experimental conditions and by different peak calling methods (GTRD “meta-cluster”) in a region spanning +/- 5,000 base pairs from the transcription start site. Transcription factors identified within these modules were also considered possible target genes to allow assessment of interactions between transcription factors and account for possible autoregulation. Transcription factors without ChIP-seq established target genes within these modules were considered only as targets for downstream analysis. The independent sets of unidirectional transcription factor to target gene interactions derived from ChIP-seq data consisted of 56,547 interactions in the magenta module and 54,621 interactions in the blue module. These ChIP-seq transcription factor to target gene interactions were input into the *monster* function as the “motif maps” and encompassed the regulatory network prior for the magenta and blue modules. In total, 81 transcription factors from 1,379 genes in the magenta module were predicted to have 106,029 target gene interactions and 104 transcription factors from 1,331 genes in the blue module were predicted to have 133,120 target gene interactions.

*Clinical Characteristics and Messenger RNA-seq (mRNA-seq) Library Preparation and Annotation for the UIC STAR Cohort*

**Peripheral blood for was obtained in heparinized collection tubes for research purposes from subjects 18 years of age and older followed at the** University of Illinois at Chicago Bernie Mac Sarcoidosis Translational Advanced Research Center (UIC STAR) with an established diagnosis of sarcoidosis in accordance with ATS/ERS/WASOG criteria [28]. Except for one subject whose diagnosis was established based on clinical history and characteristic findings on 18FDG-PET/CT, all subjects had tissue biopsies consistent with sarcoidosis. Among subjects with tissue biopsy, 72.4% (21/29) were diagnosed based on pulmonary or intrathoracic lymph node biopsy and 27.6% were diagnosed based on extrapulmonary biopsy. Subjects with history of extrathoracic biopsy had evidence of pulmonary involvement on diagnostic imaging. All subjects had available complete blood counts (CBC) with differential and CD4+ T-cell proportions by standard flow cytometry protocol performed for clinical purposes within 100 days of research sample collection. The majority (22/30, 73.3%) had clinical and research samples collected on the same day (CBC: median time between clinical and research samples = 0 days, with a range of -49 to +25 days; CD4+ T-cell percentage: median time between clinical and research samples = 0 days, with a range of -98 to +8 days). Subjects on treatment were not reported to have undergone modifications to their regimen during this time. Subjects were considered to have severe disease if there was evidence of abnormal pulmonary function (based on spirometry within 1 year of research sample collection), extensive pulmonary fibrosis on diagnostic imaging (>20%), pulmonary hypertension or other major organ involvement (ocular, neurologic, or cardiac) [12, 29-31]. Clinical characteristics for the UIC STAR cohort are summarized in **Supplemental** **Table S1C**.

Following collection of peripheral blood for research purposes, PBMCs were isolated from whole blood via a standard Ficoll-hypaque density gradient, stabilized in RNA*later*™ Stabilization Solution (Invitrogen™, Catalog #: AM7020), and stored at -80^o^C until total RNA isolation [3]. Total RNA was isolated utilizing the RiboPure™ RNA Purification Kit (Invitrogen™ Catalog #: AM1928) according to the manufacturer's protocol, quantified on the Qubit 2.0 fluorometer (*ThermoFisher Scientific Inc.*) with the Qubit™ RNA HS Assay Kit (Invitrogen™, Catalog #: Q32852). The quality of total RNA was analyzed with the RNA 6000 Pico Kit (Agilent Technologies, Catalog #:5067-1513), on the Agilent 2100 Bioanalyzer. Consistent with the ACCESS cohort, all samples had a RIN of 6 or greater. Total RNA was shipped overnight on dry ice to the BGI Group for mRNA library preparation and sequencing on the DNBSEQ-G400 platform (100 base pair paired-end reads). Upon completion of sequencing, base calls were converted into sequence data and paired-end FASTQ files were generated. The quality of sequencing reads was then assessed from FASTQ files using FastQC and followed by removal of adapter sequences by *Cutadapt* and reassessment of quality with FastQC [6, 7]. All samples demonstrated a PHRED quality score greater than 30 and were considered suitable for downstream analysis as was performed on the ACCESS cohort. FASTQ-formatted reads were aligned and quantified using *Salmon* against the human reference genome (Ensembl GRCh38.p12 release 94) indexed at the transcript level and aggregated to then be annotated at specific gene levels utilizing *Tximport* (R bioconductor package) for a total of 37,788 annotated genes [8-10]. Gene counts estimated from the *Salmon* quasi-alignments by *Tximport* were utilized for downstream analyses unless otherwise specified.

Genes from the UIC STAR cohort were matched to the uniform gene set of 12,047 genes that were commonly expressed between the ACCESS and UCSF cohorts and normalized via the trimmed mean of M-values (TMM) method and transformed to log_2_-counts per million by using *voom with sample quality weights* to down-weight outlier samples as described in the *limma* Bioconductor (**Supplemental Figure S2C**) package [18, 19]. Sarcoidosis cases in the UIC STAR cohort were grouped into lymphopenic or non-lymphopenic if CD4+ T-cell percent of PBMCs (determined by clinical flow cytometry and CBC with differential) was less than or greater than the cohort median (30.40%), respectively. Differential expression of genes within modules significantly associated with disease between the ACCESS and UCSF cohort was then assessed between lymphopenic and non-lymphopenic sarcoidosis cases based on clinical CD4+ T-cell grouping utilizing a moderated t-statistic upon adjustment for clinically relevant variables (gender, race, age, smoking status, disease duration, disease severity, and treatment status). Findings were deemed significant at a predetermined false discovery rate (FDR) of 10% (*Benjamini-Hochberg adjusted* [*BH.adj*] *p-value* <0.1) (**Supplemental** **Table S3C, Supplemental Figure S5A**) [18]. Consensus gene expression in the magenta and blue modules was ascertained between sarcoidosis cases in the ACCESS and UIC STAR cohorts (**Supplemental Figure S5B**). Overall, 30.24% of significantly over-expressed genes in the magenta module as well as 7.52% of significantly under-expressed genes in the blue module were common to both cohorts. Similarly, UCSF and UIC STAR cohorts demonstrated consensus gene expression in the magenta and blue modules (**Supplemental Figure S5C**) with 10.83% of significantly over-expressed genes in the magenta module as well as 28.63% of significantly under-expressed genes in the blue module were common to both cohorts.

*Determination of Peripheral Blood Mononuclear Cell Subpopulations by In-silico Statistical Deconvolution*

*In-silico* statistical deconvolution of gene expression data utilizing *Cibersort* was performed on every sample in all three cohorts to impute the proportions of PBMC subpopulations (**Supplemental Table S6**) [32]. To improve upon identification of immune cell proportions we utilized the Immunostates gene signature basis matrix and modified it to exclude granulocytes given that presence was unlikely since data was derived from PBMCs [33, 34]. Count data from mRNA-seq was transformed to transcripts per million (TPM) and introduced into the *Cibersort* algorithm as the “mixture file” for the ACCESS cohort [35]**. For the UCSF cohort, gene expression data as log_2_-RMA was introduced into *Cibersort* after robust multiarray average transformation. Pairwise comparisons between case-control groups and cohorts (ACCESS and UCSF) were performed by Mann-Whitney U tests to assess differences among** imputed **PBMC** subsets. To ascertain congruency of imputed cell proportions of interest with factual clinical data we utilized Pearson’s correlation under the assumption of normality and a linear relationship, Spearman’s correlation under the assumption of non-normality and a monotonic relationship, and the root mean square error (RMSE) to test **t**he association between CD4+ T-cell proportions from *in-silico* statistical deconvolution and CD4+ T-cell enumeration by standard flow cytometry protocol in the UIC STAR cohort. Given that clinical flow cytometry for monocyte subpopulations is not routinely performed at the UIC STAR Center, congruency was tested for between total imputed monocyte proportions (i.e., the sum of CD16+ monocytes, CD14+ monocytes, and M0, M1, and M2 macrophages) and clinical monocyte percent of PBMCs obtained from the CBC differential.

**References**

1. *Design of a case control etiologic study of sarcoidosis (ACCESS). ACCESS Research Group.* J Clin Epidemiol, 1999. **52**(12): p. 1173-86.

2. Kanof, M.E., Smith, P.D. and Zola, H., *Isolation of whole mononuclear cells from peripheral blood and cord blood.* Curr Protoc Immunol, 2001. **Chapter 7**: p. Unit 7 1.

3. Chomczynski, P. and Sacchi, N., *Single-Step Method of RNA Isolation by Acid Guanidium Thyocyanate-Phenol-Chloroform Extraction.* Analytical Biochemistry, 1987. **162**: p. 156-159.

4. Chomczynski, P. and Sacchi, N., *Single-step method of RNA isolation by acid guanidinium thiocyanate-phenol-chloroform extraction.* Anal Biochem, 1987. **162**(1): p. 156-9.

5. Kanof, M.E., Smith, P.D. and Zola, H., *Isolation of Whole Mononuclear Cells from Peripheral Blood and Cord Blood.* Curr Protoc Immunol, 1996. **19**(1:7.1): p. 7.1.1-7.1.7.

6. Andrews, S., *FastQC: A Quality Control Tool for High Throughput Sequence Data [Online]*. 2010.

7. Martin, M., *Cutadapt removes adapter sequences from high-throughput sequencing reads.* EMBnet.journal, 2011. **17**(1): p. 10-12.

8. Cunningham, F., Achuthan, P., Akanni, W., Allen, J., Amode, M.R., Armean, I.M., et al., *Ensembl 2019.* Nucleic Acids Res, 2019. **47**(D1): p. D745-D751.

9. Patro, R., Duggal, G., Love, M.I., Irizarry, R.A. and Kingsford, C., *Salmon provides fast and bias-aware quantification of transcript expression.* Nat Methods, 2017. **14**(4): p. 417-419.

10. Soneson, C., Love, M.I. and Robinson, M.D., *Differential analyses for RNA-seq: transcript-level estimates improve gene-level inferences.* F1000Res, 2015. **4**: p. 1521.

11. Barrett, T., Wilhite, S.E., Ledoux, P., Evangelista, C., Kim, I.F., Tomashevsky, M., et al., *NCBI GEO: archive for functional genomics data sets--update.* Nucleic Acids Res, 2013. **41**(Database issue): p. D991-5.

12. Koth, L.L., Solberg, O.D., Peng, J.C., Bhakta, N.R., Nguyen, C.P. and Woodruff, P.G., *Sarcoidosis blood transcriptome reflects lung inflammation and overlaps with tuberculosis.* Am J Respir Crit Care Med, 2011. **184**(10): p. 1153-63.

13. Team, R.C., *R: A language and environment for statistical computing*. 2016, R Foundation for Statistical Computing: Vienna, Austria.

14. Gautier, L., Cope, L., Bolstad, B.M. and Irizarry, R.A., *affy--analysis of Affymetrix GeneChip data at the probe level.* Bioinformatics, 2004. **20**(3): p. 307-15.

15. Carlson, M., *hgu133plus2.db: Affymetrix Human Genome U133 Plus 2.0 Array annotation data (chip hgu133plus2).* R package version 3.2.3. , 2016.

16. Durinck, S., Spellman, P.T., Birney, E. and Huber, W., *Mapping identifiers for the integration of genomic datasets with the R/Bioconductor package biomaRt.* Nat Protoc, 2009. **4**(8): p. 1184-91.

17. Langfelder, P. and Horvath, S., *WGCNA: an R package for weighted correlation network analysis.* BMC Bioinformatics, 2008. **9**: p. 559.

18. Ritchie, M.E., Phipson, B., Wu, D., Hu, Y., Law, C.W., Shi, W., et al., *limma powers differential expression analyses for RNA-sequencing and microarray studies.* Nucleic Acids Res, 2015. **43**(7): p. e47.

19. Liu, R., Holik, A.Z., Su, S., Jansz, N., Chen, K., Leong, H.S., et al., *Why weight? Modelling sample and observational level variability improves power in RNA-seq analyses.* Nucleic Acids Res, 2015. **43**(15): p. e97.

20. Langfelder, P., Luo, R., Oldham, M.C. and Horvath, S., *Is my network module preserved and reproducible?* PLoS Comput Biol, 2011. **7**(1): p. e1001057.

21. Monaco, G., Lee, B., Xu, W., Mustafah, S., Hwang, Y.Y., Carre, C., et al., *RNA-Seq Signatures Normalized by mRNA Abundance Allow Absolute Deconvolution of Human Immune Cell Types.* Cell Rep, 2019. **26**(6): p. 1627-1640 e7.

22. Uhlen, M., Karlsson, M.J., Zhong, W., Tebani, A., Pou, C., Mikes, J., et al., *A genome-wide transcriptomic analysis of protein-coding genes in human blood cells.* Science, 2019. **366**(6472): p. 1471/eaax9198

23. Schlauch, D., Glass, K., Hersh, C.P., Silverman, E.K. and Quackenbush, J., *Estimating drivers of cell state transitions using gene regulatory network models.* BMC Syst Biol, 2017. **11**(1): p. 139.

24. Yevshin, I., Sharipov, R., Kolmykov, S., Kondrakhin, Y. and Kolpakov, F., *GTRD: a database on gene transcription regulation-2019 update.* Nucleic Acids Res, 2019. **47**(D1): p. D100-D105.

25. UniProt, C., *UniProt: a worldwide hub of protein knowledge.* Nucleic Acids Res, 2019. **47**(D1): p. D506-D515.

26. Huang da, W., Sherman, B.T. and Lempicki, R.A., *Systematic and integrative analysis of large gene lists using DAVID bioinformatics resources.* Nat Protoc, 2009. **4**(1): p. 44-57.

27. Huang da, W., Sherman, B.T. and Lempicki, R.A., *Bioinformatics enrichment tools: paths toward the comprehensive functional analysis of large gene lists.* Nucleic Acids Res, 2009. **37**(1): p. 1-13.

28. Hunninghake, G.W., Costabel, U., Ando, M., Baughman, R., Cordier, J.F., du Bois, R., et al., *ATS/ERS/WASOG statement on sarcoidosis. American Thoracic Society/European Respiratory Society/World Association of Sarcoidosis and other Granulomatous Disorders.* Sarcoidosis Vasc Diffuse Lung Dis, 1999. **16**(2): p. 149-73.

29. Sweiss, N.J., Salloum, R., Gandhi, S., Alegre, M.L., Sawaqed, R., Badaracco, M., et al., *Significant CD4, CD8, and CD19 lymphopenia in peripheral blood of sarcoidosis patients correlates with severe disease manifestations.* PLoS One, 2010. **5**(2): p. e9088.

30. Walsh, S.L., Wells, A.U., Sverzellati, N., Keir, G.J., Calandriello, L., Antoniou, K.M., et al., *An integrated clinicoradiological staging system for pulmonary sarcoidosis: a case-cohort study.* Lancet Respir Med, 2014. **2**(2): p. 123-30.

31. Zhou, T., Zhang, W., Sweiss, N.J., Chen, E.S., Moller, D.R., Knox, K.S., et al., *Peripheral blood gene expression as a novel genomic biomarker in complicated sarcoidosis.* PLoS One, 2012. **7**(9): p. e44818.

32. Newman, A.M., Liu, C.L., Green, M.R., Gentles, A.J., Feng, W., Xu, Y., et al., *Robust enumeration of cell subsets from tissue expression profiles.* Nat Methods, 2015. **12**(5): p. 453-7.

33. Scott, M.K.D., Quinn, K., Li, Q., Carroll, R., Warsinske, H., Vallania, F., et al., *Increased monocyte count as a cellular biomarker for poor outcomes in fibrotic diseases: a retrospective, multicentre cohort study.* Lancet Respir Med, 2019. **7**(6): p. 497-508.

34. Vallania, F., Tam, A., Lofgren, S., Schaffert, S., Azad, T.D., Bongen, E., et al., *Leveraging heterogeneity across multiple datasets increases cell-mixture deconvolution accuracy and reduces biological and technical biases.* Nat Commun, 2018. **9**(1): p. 4735.

35. Jin, H., Wan, Y.W. and Liu, Z., *Comprehensive evaluation of RNA-seq quantification methods for linearity.* BMC Bioinformatics, 2017. **18**(Suppl 4): p. 117.
