# Supplementary figures and images for: Altered transcription factor targeting is associated with differential peripheral blood mononuclear cell proportions in sarcoidosis
Source: Front Immunol. 2022 Oct 13;13:848759. doi: 10.3389/fimmu.2022.848759 (PMC9608777; doi:10.3389/fimmu.2022.848759)

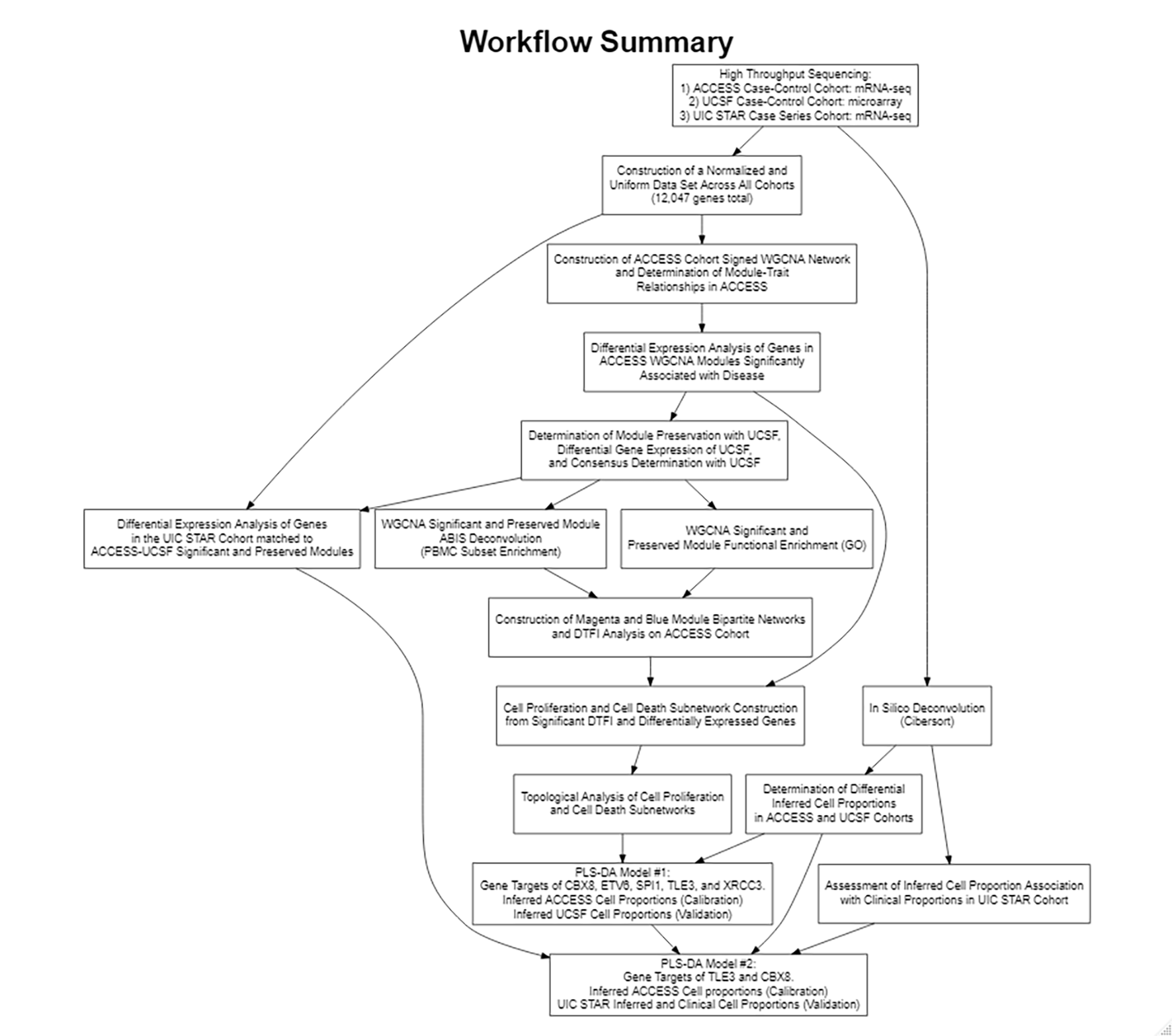

Supplement: Supplementary Figure 1 — Summarized workflow. [file Image_1.tif]

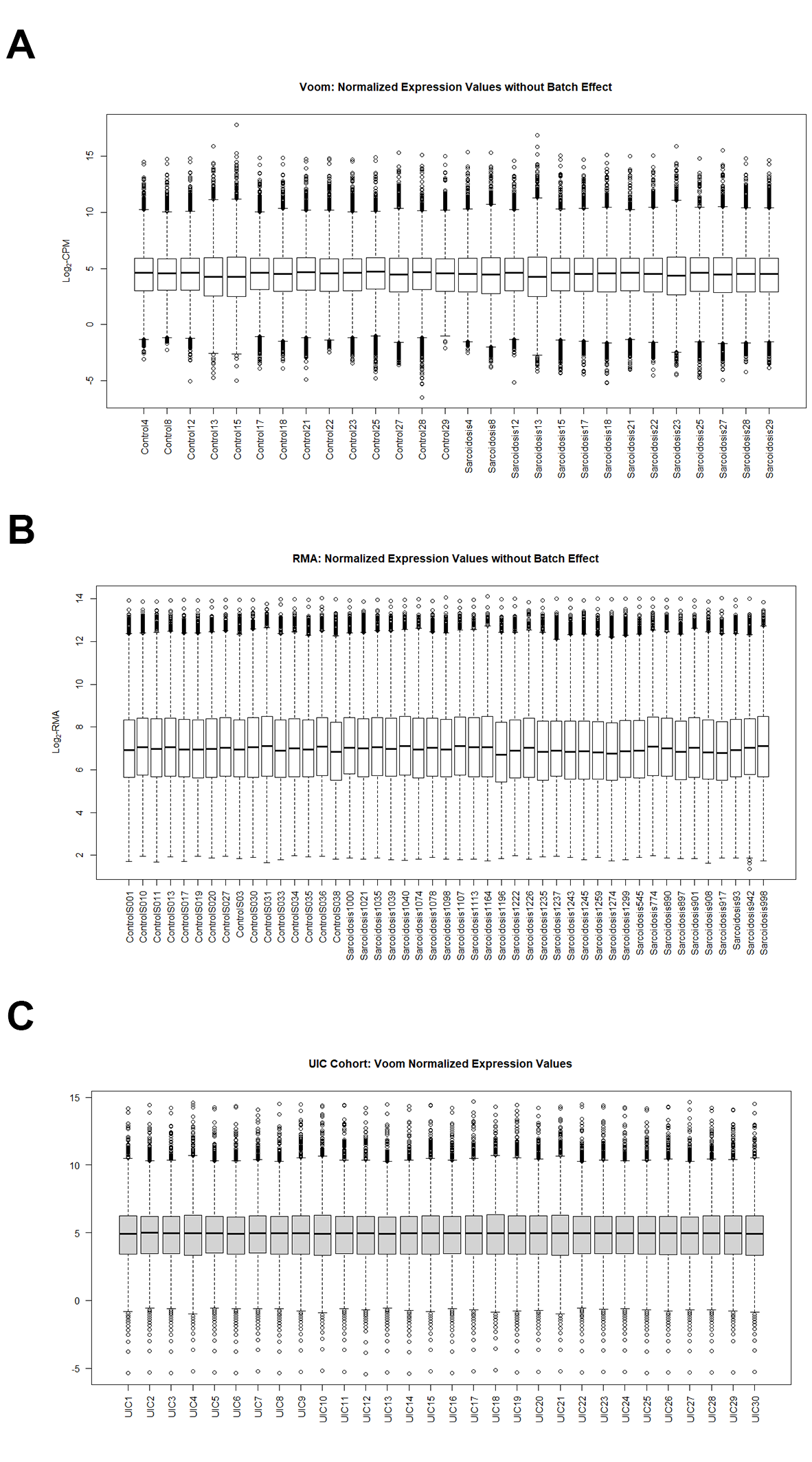

Supplement: Supplementary Figure 2 — Normalized gene expression box plots per sample. (A) Normalized gene expression in log2-counts per million utilizing the trimmed mean of M-values method for normalization and voom with sample quality weights and batch correction for the ACCESS cohort. (B) Normalized gene expression using robust multi-array average (log2-RMA) for the UCSF cohort (C) Normalized gene expression in log2-counts per million utilizing the trimmed mean of M-values method for normalization and voom with sample quality weights for the UIC STAR cohort. [file Image_2.tif]

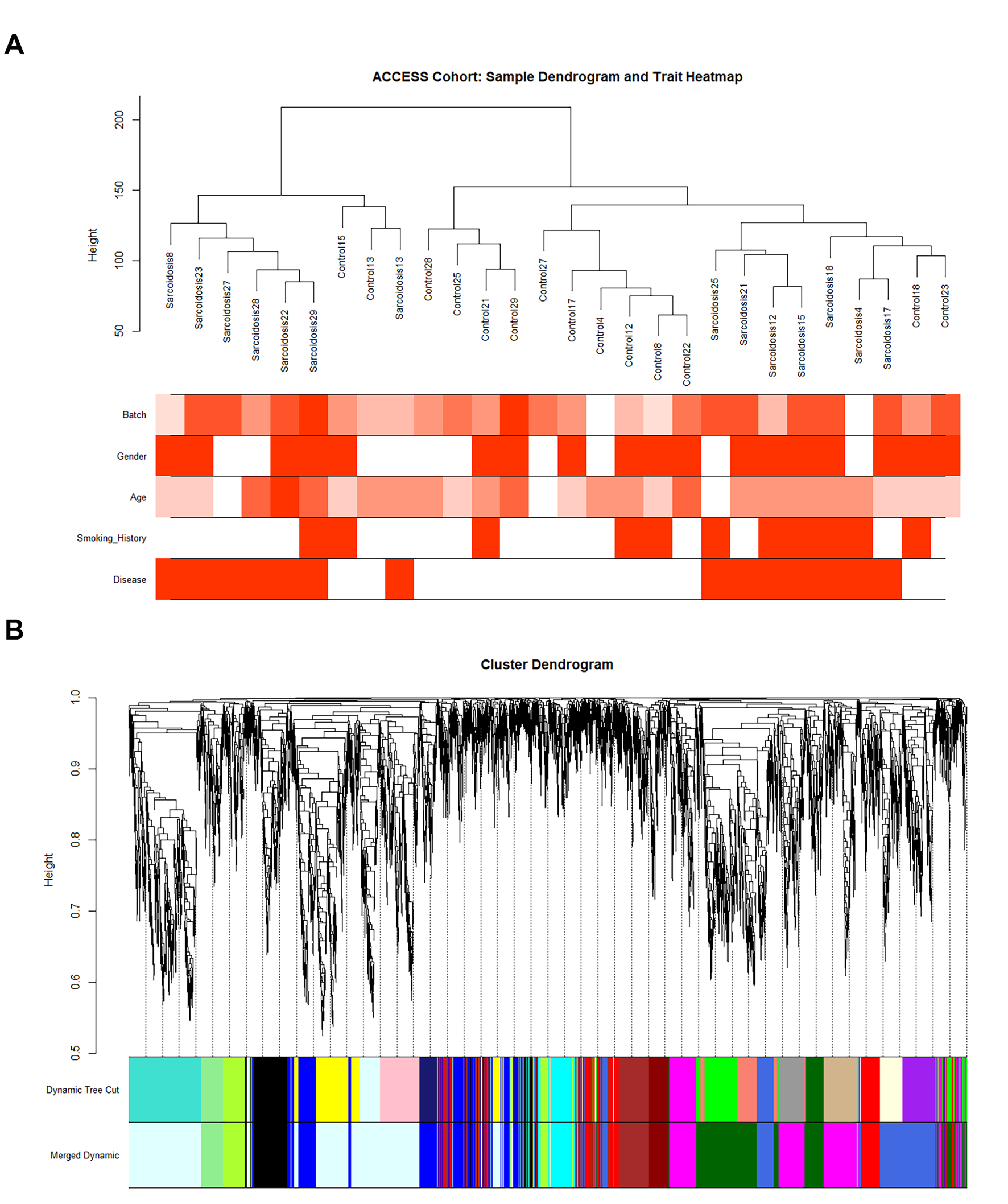

Supplement: Supplementary Figure 3 — WGCNA module construction from the ACCESS cohort. (A) Dendrogram demonstrating complete-linkage agglomerative hierarchichal clustering of subjects in the ACCESS cohort based on normalized gene expression data. Heatmap below the dendrogram is indicative of individual subject demographic characteristics in this cohort. Gender: female = red, male = white; Age: <30 years old = white, 60-69 years old = red (darker shades red are indicative of older age group); Smoking history: positive smoking history = white, never smoker = red; Disease: control = white, sarcoidosis = red. (B) Dendrogram demonstrating complete-linkage hierarchical clustering of genes with dissimilarity based on topological overlap utilizing WGCNA. Height reflects the extent of dissimilarity between genes. Genes are grouped into distinct color-coded modules as shown in the color ribbons beneath the dendrogram. Each module is separated at significant branch points in the dendrogram and consists of a set of interconnected genes identified after adaptive branch pruning (top color ribbon, “Dynamic Tree Cut”). Ultimately, 12 modules were identified after module merging at a maximum dissimilarity of 0.1 given the likelihood of high gene co-expression among closely correlated modules (bottom color ribbon, “Merged Dynamic”). [file Image_3.tif]

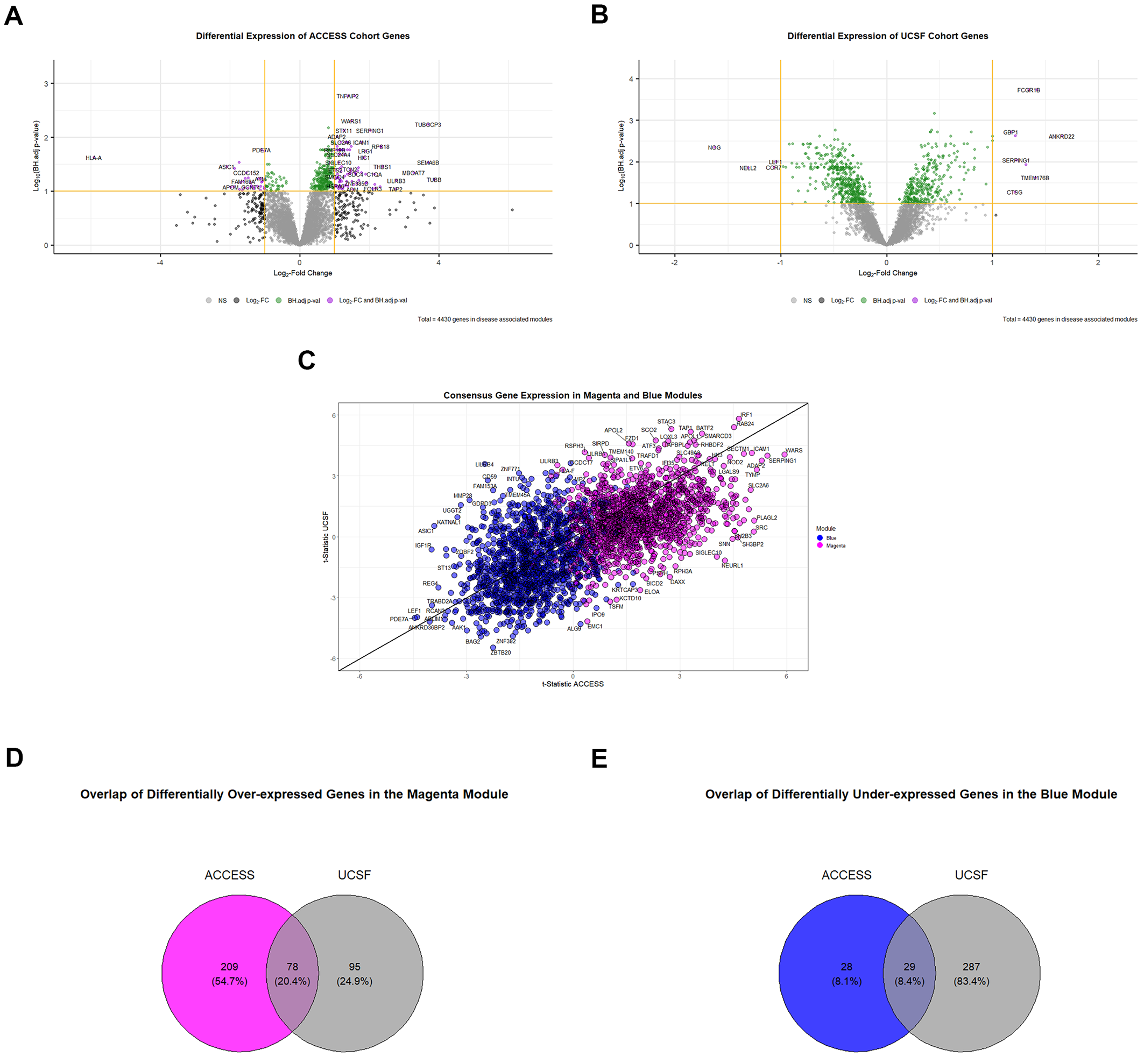

Supplement: Supplementary Figure 4 — Differential expression analysis in the ACCESS and UCSF (GSE19314) cohorts. (A, B) Volcano plots demonstrating differentially expressed genes between sarcoidosis cases and controls within WGCNA gene modules found to be significantly associated with disease (blue, brown, cyan, greenyellow, and magenta) in the ACCESS cohort (A) and the UCSF cohort (B). Significance was determined based on false discovery rate <10% (Benjamini-Hochberg adjusted [BH-adj] p-value < 0.1) utilizing the moderated t-test in the limma R-package. Horizontal gold solid line denotes BH-adj p-value of 0.1, vertical gold solid lines denote log2-fold change of 1. (C) Consensus gene expression between ACCESS and UCSF cohorts was assessed by t-statistics determined for all genes in the blue and magenta modules. (D, E) Venn diagrams demonstrate overlap of differentially over-expressed genes in the magenta (D) and differentially under-expressed genes in the blue (E) modules between cohorts. [file Image_4.tif]

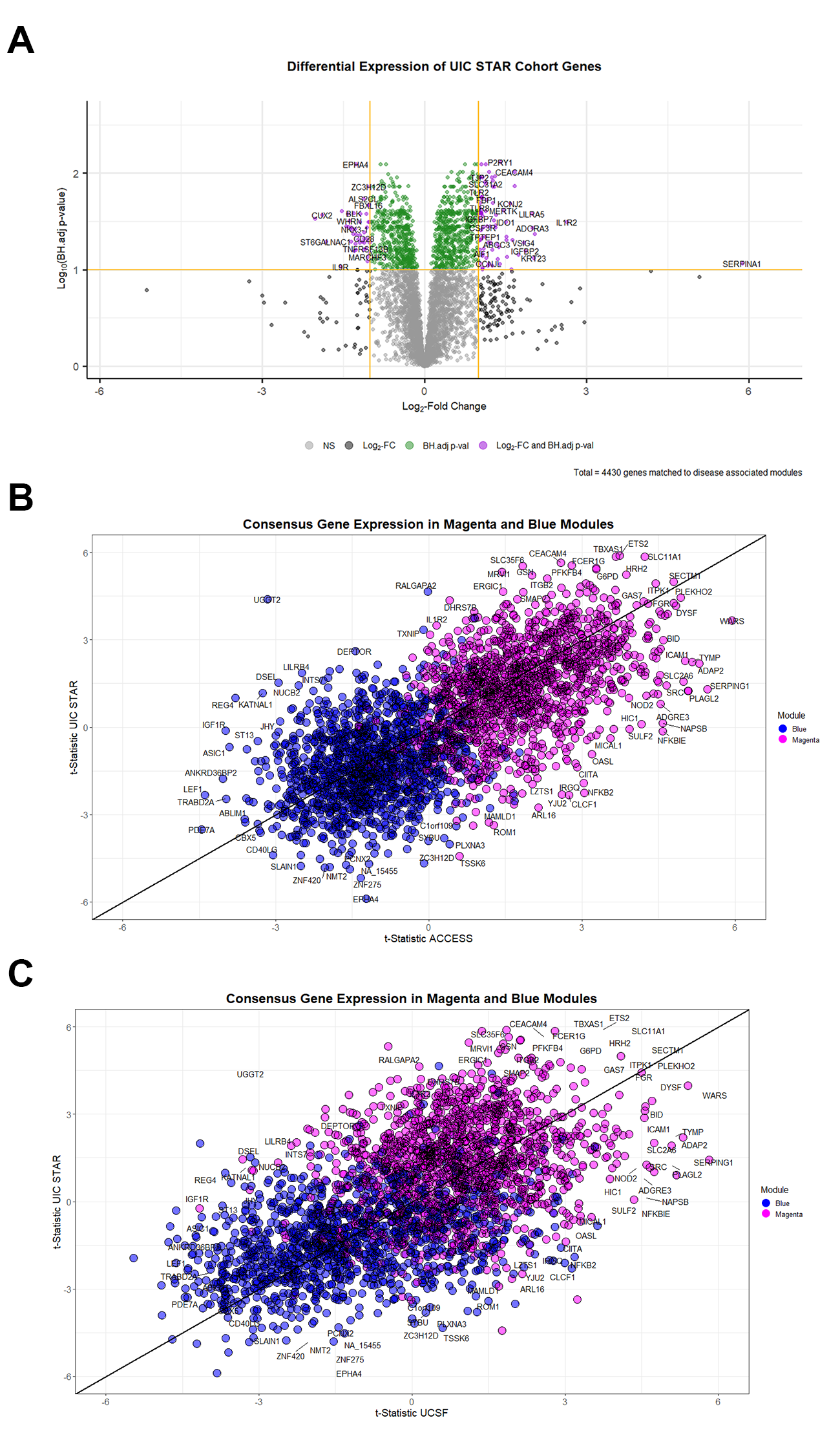

Supplement: Supplementary Figure 5 — Differential expression analysis in UIC STAR cohort. (A) Volcano plots demonstrating differentially expressed genes between lymphopenic and non-lymphopenic sarcoidosis cases based on clinical CD4+ T-cell grouping. (B) Consensus gene expression between ACCESS and UIC STAR cohorts was assessed by t-statistics determined for all genes in the blue and magenta modules. [file Image_5.tif]
